# Diaphragmatic Breathing Interfaces to Promote Relaxation for Mitigating Insomnia: Pilot Study

**DOI:** 10.2196/67000

**Published:** 2025-03-04

**Authors:** Yi-Jen Lai, Hsiao-Yean Chiu, Ko-Chiu Wu, Chun-Wei Chang

**Affiliations:** 1 Department of Interaction Design National Taipei University of Technology Taipei Taiwan; 2 School of Nursing Taipei Medical University Taipei Taiwan

**Keywords:** brief behavioral treatment for insomnia, sleep self-efficacy, mobile health, mHealth, breathing training cognitive load, attention, gamification, diaphragmatic breathing, insomnia, sleep, games, relaxation, breathing, breathing guidance, questionnaire, mental, cognition

## Abstract

**Background:**

Brief behavioral treatment for insomnia is an effective short-term therapy focusing on stimulus control and sleep restriction to enhance sleep quality. As a crucial part of this therapy, diaphragmatic breathing is often recommended when patients fail to fall asleep within 30 minutes. With the rise of health apps and gamification, these tools are increasingly seen as effective ways to boost self-efficacy and user engagement; however, traditional games tend to increase attention, which can negatively impact sleep and contradicts the aim of sleep therapy. This study thus explored the potential for gamification techniques to promote relaxation without disrupting sleep processes.

**Objective:**

The study developed 4 breathing guidance mechanisms, ranging from concrete to abstract: number countdown, zoom-in/out, up/down, and color gradients. The objective was to explore the relationship between game mechanics, cognitive load, relaxation effects, and attention as well as to understand how different designs impact users with varying levels of insomnia.

**Methods:**

The study was conducted in 2 phases. The first phase involved a questionnaire on the 4 guidance mechanisms. In the second phase, 33 participants classified by insomnia severity completed a Sleep Self-Efficacy Scale. They then engaged in 5 minutes of diaphragmatic breathing using each of the 4 interfaces. Relaxation effects were measured using heart rate variability via a smartwatch, attention and relaxation levels via an electroencephalogram device, and respiratory rate via a smartphone. Participants also completed the Game Experience Questionnaire and NASA Task Load Index, followed by user interviews.

**Results:**

The results indicated that competence, immersion, and challenge significantly influenced cognitive load. Specifically, competence and immersion reduced cognitive load, while challenge, negative affect, and positive affect were correlated with relaxation. Negative affect showed a positive correlation with the mean root mean square of successive differences, while positive affect exhibited a negative correlation with the mean root mean square of successive differences. Cognitive load was found to affect both relaxation and attention, with a negative correlation between mental demand and attention and a positive correlation between temporal demand and respiratory rate. Sleep self-efficacy was negatively correlated with temporal demand and negative affect and positively correlated with competence and immersion.

**Conclusions:**

Interfaces offering moderate variability and neither overly abstract nor too concrete guidance are preferable. The up/down interface was most effective, showing the best overall relaxation effect. Conversely, the number countdown interface was stress-inducing, while the zoom-in/out interface had a significant impact on insomnia-related issues, making them less suitable for insomnia-related breathing exercises. Participants showed considerable variability in their response to the color gradient interface. These findings underscore the importance of carefully considering game design elements in relaxation training. It is essential that breathing guidance designs account for the impact of the game experience to effectively promote relaxation in users.

## Introduction

### Background

Insomnia is an increasingly severe issue, with approximately 30% of the population experiencing related symptoms [1]. It is characterized by difficulty in falling asleep, maintaining sleep, or waking up too early and being unable to return to sleep for at least 3 nights a week. This definition is based on the *Diagnostic and Statistical Manual of Mental Disorders, Fifth Edition* criteria. Acute insomnia lasts for 1 month, while chronic insomnia persists for over 3 months [2]. Cognitive behavioral therapy for insomnia (CBT-I) is the most common behavioral treatment [3]. CBT-I typically involves 4 to 8 sessions focusing on changing thoughts, emotions, and behaviors related to sleep [4]. Derived from CBT-I, the brief behavioral treatment for insomnia (BBTI) condenses the process into 4 weeks, focusing on sleep restriction and stimulus control [5]. Compared with CBT-I, BBTI requires less time and resources, making it accessible to a wider range of patients.

Many people attempt to compensate for poor sleep patterns by spending excessive time in bed, which often increases anxiety about sleep. Sleep restriction reduces the time spent in bed to approximately match the actual sleep time, while stimulus control helps to reestablish the connection between the bed and sleep, reducing anxiety and frustration [4]. Relaxation training is an important adjunct to insomnia treatment, particularly diaphragmatic breathing. Slow breathing can regulate the autonomic nervous system, increasing heart rate variability (HRV) and electroencephalogram (EEG) α power, leading to a state of calm and relaxation [6].

A variety of health apps focus on insomnia treatment, such as sleep diaries; however, there are few relaxation training apps specifically designed for insomniacs. Research on breathing relaxation has often focused on enhancing breathing techniques and analyzing different breathing guidance designs. One study examined 3 types of breathing guidance designs, including voice-only guidance and 2 combinations of voice and visuals. The results showed that wave visualization designs outperformed voice-only designs in improving both objective breathing performance and user experience [7]. Similarly, Faust-Christmann et al’s [8] study confirmed that visual guidance effectively helps users achieve breathing goals and suggested further exploration of cognitive levels during training sessions as well as other psychophysiological data. A study on breathing training in a virtual reality environment revealed that users’ progress was primarily concentrated in the first 2 sessions. Moreover, participants’ subjective impression of progress was found to be more critical than objective improvements, as it significantly enhanced their motivation and willingness to participate [9].

The main challenge for health apps is ensuring long-term regular use. In this endeavor, understanding user needs is crucial [10]. Users initially engage with health apps to meet health needs, but over time, enjoyment becomes increasingly important [11]. Gamification, which involves adding interactive elements to nongame contexts [12], is often used in health apps to attract patients through engaging interactions, promote adherence, and facilitate positive behavioral change [13].

Albert Bandura proposed the theory of self-efficacy, which refers to the ability to perceive oneself as being able to achieve a goal. This theory is used to measure the degree or strength of an individual’s belief in their ability to accomplish a task [14]. Feedback from games can enhance self-efficacy, thereby affecting user participation and persistence in activities [15]. Virtual reality biofeedback games can help to increase self-efficacy and reduce stress; there is evidence that higher self-efficacy is correlated with an increased ability to relax postgaming [16]. Enhanced self-efficacy also supports adherence to BBTI, while low self-efficacy is associated with poorer compliance with stimulus control therapy and sleep hygiene [17].

Sleep self-efficacy, an extension of general self-efficacy, measures an individual’s perceived ability to influence their own sleep behavior to achieve healthy sleep. The Sleep Self-Efficacy Scale, which comprises 9 items, assesses this perception [18]. As insomnia severity, poor health, depression, and misconceptions about sleep increase, sleep self-efficacy decreases [19]. Both general self-efficacy and sleep self-efficacy are predictors of sleep health, with higher levels associated with better sleep quality, regularity, and scheduling [20].

Sweller [21] proposed the concept of cognitive load. It assumes that human cognitive resources are limited and that excessive cognitive load may affect the effectiveness of learning and task performance. Increased cognitive load before sleep can lead to arousal and longer sleep latency, affecting sleep quality [22]. Attention is also closely linked to sleep; the cognitive model of insomnia suggests that insomniacs often over-concern themselves with sleep, focusing attention on perceived sleep threats, such as bodily sensations or environmental factors, which can increase arousal and anxiety, leading to misconceptions that disrupt sleep [23]. Sleep should be an unconscious process [24]. Conscious attempts to fall asleep may disrupt sleep, and insomniacs are more likely than good sleepers to have difficulty falling asleep due to anxiety and environmental disruptions. Insomniacs are also more likely to think about not being able to fall asleep or things that have happened during the day before they go to bed. Using the NASA Task Load Index (NASA-TLX) scale to assess the association between serious gaming and cognitive load, Sevcenko [25] found that both task complexity and scene difficulty affected participants’ cognitive load and impacted gaming performance.

With the proliferation of health apps and medical gamification, gamifying relaxation training has become an effective way to enhance training outcomes. Many apps target breathing training; however, few focus on patients with insomnia and presleep use, as traditional games often increase attention or excitement, which can interfere with sleep.

### Objectives

This study aims to investigate how different breathing guidance mechanisms impact relaxation and whether these effects are related to the severity of insomnia and sleep self-efficacy. The selected variables were cognitive load, attention, gaming experience, and relaxation levels achieved through breathing guidance.

We hypothesize that concrete breathing guidance interfaces are more effective than abstract guidance in reducing respiration rates. Abstract breathing guidance interfaces are associated with lower cognitive load compared with concrete interfaces. In addition, we hypothesized that the positive dimensions of the Game Experience Questionnaire (GEQ; eg, competence, immersion, flow, and positive affect) are negatively associated with cognitive load, while the negative dimensions (eg, frustration, challenge, and negative affect) are positively associated with cognitive load.

## Methods

### Four Guidance Mechanisms

In this study, various breathing-related games were categorized into 4 guidance mechanisms: countdown, zoom-in/out, up/down movements, and color gradients. These methods range from concrete to abstract guidance and from small to large visual variations.

1. Countdown: Guidance is provided through a numerical countdown on the screen.
2. Zoom-in/out: Guidance is provided through the enlargement and reduction of a graphic, with enlargement indicating inhalation and reduction indicating exhalation.
3. Up/down movements: Guidance is provided through the vertical movement of the screen, with upward movement indicating inhalation and downward movement indicating exhalation.
4. Color gradients: Guidance is provided through changes in the color gradient on the screen.

### Interface Design

#### Main Visuals

**Figure 1:**
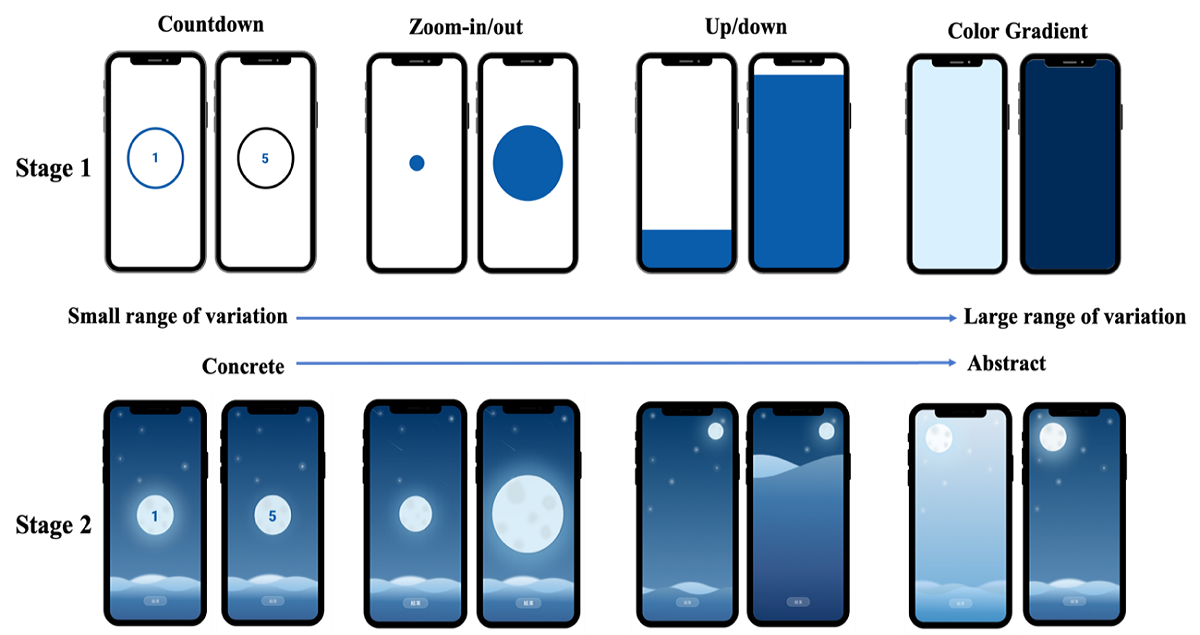
Prototypes of interface design for 4 guidance mechanisms.

The interface for relaxation training through diaphragmatic breathing features nighttime ocean waves as the main visual (Figure 1). The color blue is associated with many positive emotions, including relaxation, calmness, happiness, and comfort [26], because blue is often linked to natural elements such as the ocean, water, and sky, which evoke feelings of relaxation and tranquility. It has also been found that blue is the most favored color among Asian populations [27]. In addition, images of natural landscapes produce higher levels of relaxation and are less stimulating than images of urban landscapes [28]. Among images of deserts, forests, snow, and water, researchers found images with water elements were the most relaxing [25].

#### Music

The game uses white noise from ocean waves combined with relaxing music as background audio. White noise has been proven to improve sleep quality by reducing environmental noise and inducing relaxation [29]. Compared with natural white noise, natural soundscapes, and ambient music, listening to instrumental music or instrumental music mixed with natural soundscapes has been found to be the most relaxing [27]. The combination of natural white noise and music enhances the sense of immersion and stimulates imagination. This allows participants to visualize the natural environment in their mind, creating a sense of being in the natural environment, while the diversity of the music provides a better listening experience.

#### Guidance Mechanism

Diaphragmatic breathing exercises are conducted in 5-minute sessions to ensure accurate HRV measurements [30]. The interface guides the user through 6 breaths per minute, as 6 breaths per minute achieve the highest HRV [31]. It also controls vagal activity, which helps insomniacs maintain a stable sleep cycle and shorten the latency to sleep through regular breathing, improving the quality of sleep [32].

### Measurement

#### Overview

In the experiment, we used a Garmin smartwatch to measure HRV and paired it with a BrainLink dynamic EEG headset (60 Hz) to assess relaxation and attention levels during gameplay, relaxation and attention scores range from 0 to 100. The Google Fit mobile app was used to measure breathing rates before and after the game sessions. All these devices were noninvasive and collected physiological signals from users during the relaxation game, which were then used for subsequent statistical analysis.

#### Breathing Rate

The normal resting breathing rate for an adult is between 12 and 20 breaths per minute. However, breathing at a rate of 6 breaths per minute has been shown to achieve the highest HRV. Therefore, this study measured the changes in breathing rates before and after using the interface to determine which guiding method is most effective in reducing breathing rates.

#### Relaxation Level

HRV is an indicator of relaxation. The standard deviation of normal to normal intervals (SDNN) is used for assessing overall variability and heart health, whereas the root mean square of successive differences (RMSSD) is more influenced by the parasympathetic nervous system and is a suitable method for quantifying short-term HRV [33,34]. α power is closely related to psychological relaxation states. α power activity increases significantly when the individual is in a relaxed state. EEG data can also reflect specific psychological and emotional states [35-38]. Using a smartwatch to measure heart rate and HRV provides a direct reflection of physiological states, while EEG data reveal psychological relaxation activities and changes in attention during the relaxation process.

### Questionnaire

#### Insomnia Severity Index

This scale assessed the severity of insomnia [39] in participants. Scores ranging from 0 to 7 indicate “no insomnia”; scores ranging from 8 to 14 indicate “mild insomnia”; and scores of 15 and above indicate “moderate to severe insomnia.”

#### NASA Task Load Index

This scale evaluated the cognitive load [40] experienced by users under different breathing guidance methods during the diaphragmatic breathing game. It consists of 6 items measuring mental, physical, and temporal demands as well as performance, effort, and frustration. Scores range from 0 to 100, with lower scores indicating better performance or lower subjective load in each dimension.

#### AttrakDiff Mini

AttrakDiff [41] is a tool used to assess user experience, analyzing the pragmatic quality (PQ), hedonic quality-stimulation (HQ-S), hedonic quality-identity (HQ-I), and attractiveness of interactive products. The full scale contains 28 items, while the AttrakDiff Mini reduces this to 10 items. This study used a 7-point scale (–3: negative, 3: positive) to measure the PQ, HQ-S, HQ-I, and attractiveness of the interfaces.

#### Sleep Self-Efficacy Scale

This scale [18] consisted of 9 items assessing participants’ confidence in their ability to sleep. The higher the total score, the more confident participants are in their ability to sleep.

#### GEQ

The GEQ [42] assessed players’ game experiences, containing 33 items covering 7 dimensions: competence, sensory and imaginative immersion, flow, tension, challenge, negative affect, and positive affect. This study used the GEQ to evaluate subjective game experiences across different game guidance methods and to analyze which aspects of the game influence relaxation.

### Experimental Procedure

The anonymous questionnaire items covered age, gender, work status, past experience with relaxation apps, and the Insomnia Severity Index (ISI) to assess participants’ insomnia severity. Afterward, the participants watched videos of the 4 different breathing modalities following sequences: countdown, zoom-in/out, up/down movements, and color gradients, each of which was a 30-second video of each of the 4 interfaces, and then filled out the NASA-TLX workload scale and the AttrakDiff Mini scale to assess the cognitive load and experience of each modality. This process was repeated 4 times, once for each breathing guidance method. Based on the results and feedback from the first phase, the diaphragmatic breathing interfaces were refined and improved.

**Figure 2:**
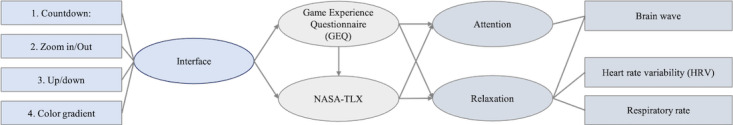
Second phase: correlation between questionnaires and physiological signals. NASA-TLX: NASA Task Load Index.

In the second phase, participants were recruited to perform 5-minute diaphragmatic breathing sessions. Participants included individuals with no insomnia, mild insomnia, and severe insomnia. They first completed a basic information form, which included demographic data, the ISI, and the Sleep Self-Efficacy Scale. Participants wore a Garmin smartwatch to collect HRV and a BrainLink Dynamic Brainwave Instrument to measure mental relaxation and attention as well as used a cell phone app, Google Fit, to measure respiratory rate before and after the experiment. Using a Latin square design to randomize the order, participants performed diaphragmatic breathing exercises with 4 different interfaces, each for 5 minutes. After each session, participants completed the NASA-TLX scale and the GEQ to evaluate the cognitive load and user experience of each interface. Upon completing all sessions, semistructured interviews were conducted to gather feedback on the design of the game mechanisms and preferences as well as explore possible reasons for relaxation during the game. This experimental design allowed for a comprehensive evaluation of how participants with varying insomnia severity responded to the 4 breathing guidance methods, using both physiological signals and subjective assessments to identify the most effective relaxation training methods. Figure 2 shows the correlogram of the second stage of the experiment, which assessed the response of participants with different severities of insomnia to the 4 types of breathing guidance, and identified the most effective relaxation training method through physiological signals and subjective ratings.

### Ethical Considerations

The study was approved by the TMU-Joint Institutional Review Board of Taipei Medical University and its affiliated hospital under the general review case number N20243070. Before participating in the second phase of the experiment, all participants provided written informed consent, confirming their full understanding of the study procedures and their agreement to participate. Additionally, participants were asked whether they had any symptoms listed in the exclusion criteria. All data collected were anonymized and deidentified to ensure the privacy and confidentiality of participants. No personally identifiable information was retained or shared during or after the study. Participants were provided with a gift voucher for a convenience store as a reward for their participation. Even if participants were unable to complete the experiment for any reason, the gift voucher was still given, and there was no requirement to return it. No images of individual participants or identifiable features are included in the paper or Multimedia Appendix 1.

## Results

### Overview

**Figure 3:**
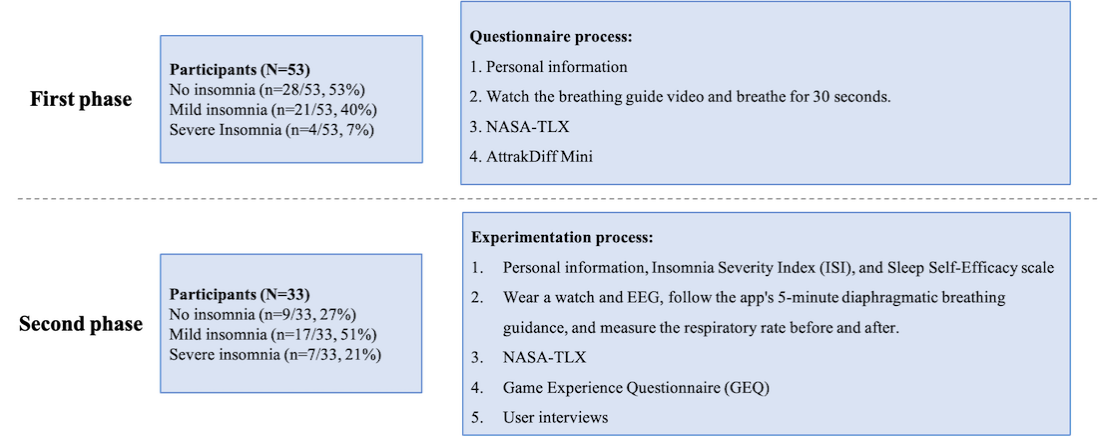
Number of participants and experimental procedure for 2-phase study. EEG: electroencephalogram; NASA-TLX: NASA Task Load Index.

In the first phase of the breathing guidance prototype survey, conducted between January and February 2024, a total of 53 valid questionnaires were collected. The participants ranged in age from 18 to 65 years and included both genders. Among them, 28 (52.8%) participants had no insomnia, 21 (36.9%) participants had mild insomnia, and 4 (7.5%) participants had severe insomnia. In the second phase of the interface design experiment, conducted from May to July 2024, 33 participants were recruited, ranging in age from 20 to 65 years, including both genders, without excluding any participants. Among these participants, 9 (27.3%) participants had no insomnia, 17 (51.5%) participants had mild insomnia, and 7 (21.2%) participants had severe insomnia. The experimental steps and the number of participants are shown in Figure 3.

### Changes in Breathing Rate

As shown in Table 1, breathing rates decreased the least (1.13) after participants used the zoom-in/out interface. Breathing rates decreased respectively by 1.41 and 1.53 after participants used the countdown interface and the up/down interface. Breathing rates decreased the most (1.75) after participants used the color gradient interface. Paired sample *t* tests were used to analyze breathing rates before and after the intervention. There was a significant difference in breathing rates before and after participants used the up/down interface (*P*=.04) and the color gradient interface (*P*=.02). This indicates that these 2 guidance methods can effectively regulate breathing frequency through relaxation training.

**Table 1:** Comparison of breathing rates before and after diaphragmatic breathing training.

Interface style	Mean (pretraining)	Mean (posttraining)	Mean difference (SD)	95% CI	*t* value	Significance (2-tailed)
Countdown	13.47	12.06	1.41 (4.063)	–0.059 to 2.871	1.958	0.059
Zoom-in/out	12.91	11.78	1.13 (4.549)	–0.515 to 2.765	1.399	0.172
Up/down	13.00	11.47	1.53 (3.992)	0.092 to 2.970	2.170	0.038
Color gradients	13.34	11.59	1.75 (3.976)	0.317 to 3.183	2.490	0.018

### Interface Measurement

**Figure 4:**
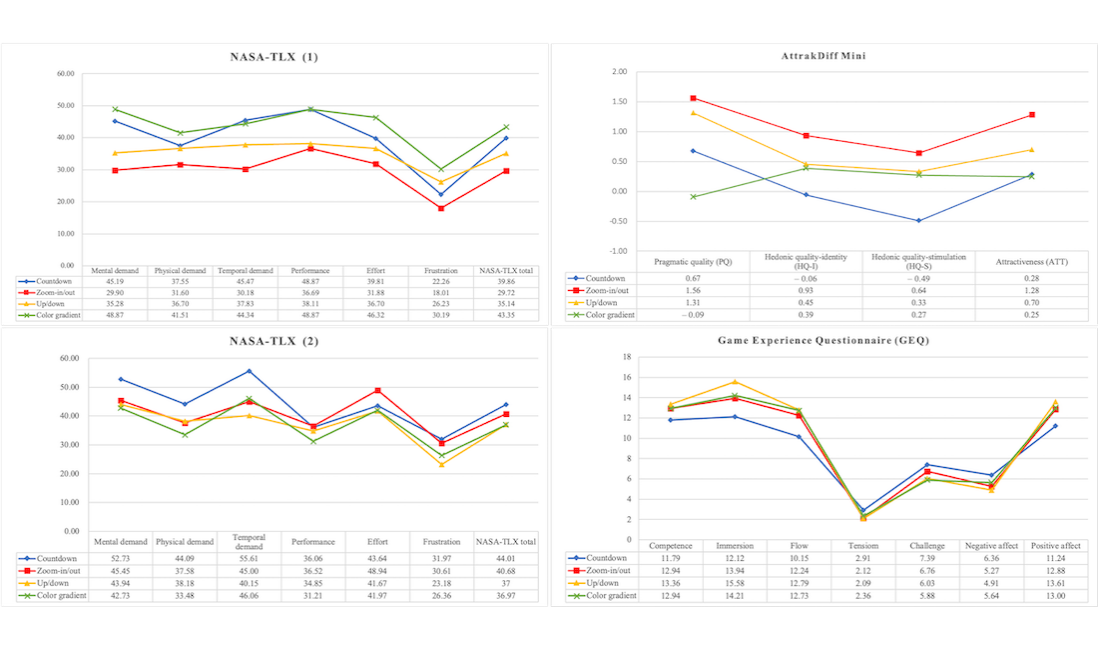
Mean values of NASA-TLX (measuring cognitive load), Attrakdiff Mini (assessing user experience), and the Game Experience Questionnaire (evaluating gaming experience) across 4 interfaces. NASA-TLX: NASA Task Load Index.

Figure 4 describes the average cognitive load, use experience, and gaming experience of the 4 pretest interventions. According to the NASA-TLX (1) results from the first phase of the experiment, the cognitive load induced by the countdown interface and the up/down interface was 39.86 and 35.14, respectively. The zoom-in/out interface induced the lowest cognitive load at an average of 29.72, while the color gradient interface induced the highest cognitive load at an average of 43.35. The AttrakDiff Mini results showed that overall satisfaction was the lowest for the countdown interface, particularly in HQ-I and HQ-S scores, which were negative. The zoom-in/out interface had the highest overall satisfaction, while the color gradient interface had the lowest PQ among the 4 interfaces.

In the second phase of the experiment, the NASA-TLX (2) results showed that the countdown interface induced the highest overall cognitive load, with high scores in psychological, physical, time, and frustration loads. The zoom-in/out interface induced the second-highest cognitive load, with the highest scores in performance and effort, indicating that this interface led to poorer performance and required more effort. The up/down interface induced the second lowest overall cognitive load, with the lowest scores in time pressure, effort, and frustration. The color gradient interface induced the lowest cognitive load, with the lowest scores in psychological, physical, and performance loads, indicating that it was less likely to cause psychological and physical strain.

### Physiological Measurements

Table 2 illustrates the results of relaxation measurements from the second phase. The data included the average respiratory rate per minute after 5 minutes of diaphragmatic breathing, average heart rate, average SDNN, and average RMSSD measured during diaphragmatic breathing using a smartwatch as well as relaxation level, relaxation stability, attention, and attention stability measured using an EEG device. For the up-down interface, the respiratory rate and average heart rate were the lowest while the average RMSSD was the highest, indicating that the up-down interface provides better breathing guidance and relaxation effects. Additionally, the zoom-in/out interface showed the highest psychological relaxation, the up-down interface had the highest relaxation stability and attention stability, and the color gradient interface had the highest attention.

**Table 2:** Physiological and psychological measurements of 4 interfaces.

Interface style and physiological signal			Minimum		Maximum		Mean (SD)
**Countdown**							
	Breathing rate (bpm)	6		22		12.06 (3.98)	
	Mean heart rate (bpm)	62		107		81.58 (10.12)	
	Mean SDNN^a^ (ms)	9.89		90.41		45.74 (16.09)	
	Mean RMSSD^b^ (ms)	15.75		74.84		29.37 (12.08)	
	Relaxation	28		66		52.85 (7.95)	
	Relaxation stability	42		77		52.15 (6.15)	
	Attention	33		72		51.94 (9.09)	
	Attention stability	20		77		55.79 (12.46)	
**Zoom-in/out**							
	Breathing rate (bpm)	6		24		11.78 (4.76)	
	Mean heart rate (bpm)	66		110		81.03 (10.25)	
	Mean SDNN (ms)	29.77		102.18		50.43 (16.88)	
	Mean RMSSD (ms)	15.79		63.29		31.55 (11.28)	
	Relaxation	42		71		56.30 (8.60)	
	Relaxation stability	43		60		51.42 (4.02)	
	Attention	34		68		52.12 (7.47)	
	Attention stability	31		79		57.00 (10.70)	
**Up/down**							
	Breathing rate (bpm)	6		20		11.47 (4.12)	
	Mean heart rate (bpm)	64		108		80.36 (10.57)	
	Mean SDNN (ms)	29.70		86.17		50.22 (16.24)	
	Mean RMSSD (ms)	11.85		60.28		33.06 (12.21)	
	Relaxation	34		68		55.48 (7.76)	
	Relaxation stability	45		62		52.82 (4.33)	
	Attention	34		73		52.24 (9.57)	
	Attention stability	44		73		59.24 (8.39)	
**Color gradients**							
	Breathing rate (bpm)	6		24		11.59 (4.85)	
	Mean heart rate (bpm)	58		113		80.94 (11.13)	
	Mean SDNN (ms)	26.17		107.45		50.56 (16.67)	
	Mean RMSSD (ms)	12.29		77.00		32.47 (14.08)	
	Relaxation	43		69		55.70 (6.79)	
	Relaxation stability	41		67		50.88 (5.41)	
	Attention	37		73		52.33 (9.95)	
	Attention stability	26		77		57.85 (10.03)	

^a^SDNN: standard deviation of normal to normal interval.

^b^RMSSD: root mean square of successive differences.

### Impact of Gaming Experience on Cognitive Load

Tables 3-5 present the results of multivariate analysis of variance (MANOVA) analysis. Each participant completed the questionnaire 4 times, corresponding to 4 different interfaces. Therefore, we applied the Bonferroni correction, which adjusts the significance level for each individual test by dividing α (0.05) by 4, resulting in a new *P* value of .0125.

**Table 3:** Influence and correlations among gaming experience and cognitive load.

Source and dependent variable			*F* test (*df*)		*P* value		Correlation		Significance (2-tailed test)
**Competence**									
	Mental demand	12.639 (1, 131)		.001^a^		–0.301		0.000^a^	
	Physical demand	2.836 (1, 131)		.10		–0.190		0.029	
	Temporal demand	4.660 (1, 131)		.03		–0.395		0.000^a^	
	Performance	9.902 (1, 131)		.002^a^		–0.547		0.000^a^	
	Effort	3.050 (1, 131)		.08		–0.367		0.000^a^	
	Frustration	10.168 (1, 131)		.002^a^		–0.561		0.000^a^	
**Immersion**									
	Mental demand	9.523 (1, 131)		.003^a^		–0.240		0.006^a^	
	Physical demand	5.606 (1, 131)		.02		–0.116		0.186	
	Temporal demand	0.429 (1, 131)		.51		–0.203		0.019	
	Performance	1.301 (1, 131)		.26		–0.259		0.003^a^	
	Effort	6.385 (1, 131)		.013		–0.278		0.001^a^	
	Frustration	1.592 (1, 131)		.21		–0.216		0.013	
**Challenge**									
	Mental demand	0.226 (1, 131)		.64		0.303		0.000^a^	
	Physical demand	3.592 (1, 131)		.06		0.410		0.000^a^	
	Temporal demand	3.241 (1, 131)		.07		0.422		0.000^a^	
	Performance	5.649 (1, 131)		.02		0.441		0.000^a^	
	Effort	17.471 (1, 131)		<.001^a^		0.586		0.000^a^	
	Frustration	8.012 (1, 131)		.005^a^		0.303		0.000^a^	
**Positive affect**									
	Mental demand	7.264 (1, 131)		.008^a^		–0.130		0.138	
	Physical demand	4.187 (1, 131)		.04		0.017		0.847	
	Temporal demand	1.578 (1, 131)		.21		–0.224		0.010^a^	
	Performance	1.893 (1, 131)		.17		–0.390		0.000^a^	
	Effort	0.677 (1, 131)		.41		–0.198		0.023	
	Frustration	0.131 (1, 131)		.72		–0.357		0.000^a^	

^a^*P*<.0125.

**Table 4:** Impact of cognitive load on relaxation.

Source and dependent variable		*F* test (*df*)	*P* value	Correlation	Significance (2-tailed test)
**Mental demand**					
	Breathing rate (bpm)	0.100 (1, 126)	.75	0.077	0.393
	Mean heart rate (bpm)	0.315 (1, 126)	.58	–0.094	0.294
	Mean SDNN^a^ (ms)	0.384 (1, 126)	.54	0.185	0.038
	Mean RMSSD^b^ (ms)	0.034 (1, 126)	.85	0.101	0.260
	Relaxation	2.599 (1, 126)	.11	0.000	0.996
	Relaxation stability	0.050 (1, 126)	.82	0.027	0.763
	Attention	13.395 (1, 126)	<.001^c^	–0.308	0.000^c^
	Attention stability	0.343 (1, 126)	.56	0.146	0.101
**Temporal demand**					
	Breathing rate (bpm)	7.900 (1, 126)	.006^c^	0.245	0.005^c^
	Mean heart rate (bpm)	0.281 (1, 126)	.60	0.004	0.968
	Mean SDNN (ms)	0.017 (1, 126)	.90	0.073	0.417
	Mean RMSSD (ms)	0.082 (1, 126)	.78	0.017	0.850
	Relaxation	3.491 (1, 126)	.06	0.159	0.074
	Relaxation stability	0.429 (1, 126)	.51	–0.017	0.853
	Attention	1.169 (1, 126)	.28	–0.072	0.423
	Attention stability	0.598 (1, 126)	.44	0.116	0.194

^a^SDNN: standard deviation of normal to normal interval.

^b^RMSSD: root mean square of successive differences.

^c^*P*<.0125.

**Table 5:** Impact of gaming experience on relaxation.

Source and dependent variable			*F* test (*df*)		*P* value		Correlation		Significance (2-tailed test)
**Flow**									
	Breathing rate (bpm)	0.015 (1, 126)		.90		–0.083		0.355	
	Mean heart rate (bpm)	7.926 (1, 126)		.006^a^		–0.081		0.367	
	Mean SDNN^b^ (ms)	8.132 (1, 126)		.005^a^		0.063		0.481	
	Mean RMSSD^c^ (ms)	11.171 (1, 126)		.001^a^		0.006		0.946	
	Relaxation	0.058 (1, 126)		.81		–0.021		0.814	
	Relaxation stability	0.000 (1, 126)		.99		0.096		0.280	
	Attention	7.420 (1, 126)		.007^a^		–0.076		0.393	
	Attention stability	0.001 (1, 126)		.97		0.026		0.769	
**Tension**									
	Breathing rate (bpm)	0.049 (1, 126)		.83		0.227^a^		0.010^a^	
	Mean heart rate (bpm)	6.938 (1, 126)		.01^a^		0.047		0.599	
	Mean SDNN (ms)	1.746 (1, 126)		.19		0.025		0.778	
	Mean RMSSD (ms)	2.423 (1, 126)		.12		0.087		0.332	
	Relaxation	0.591 (1, 126)		.44		0.150		0.093	
	Relaxation stability	1.936 (1, 126)		.17		–0.251^a^		0.004^a^	
	Attention	0.853 (1, 126)		.36		–0.022		0.809	
	Attention stability	0.347 (1, 126)		.56		0.010		0.914	
**Challenge**									
	Breathing rate (bpm)	4.301 (1, 126)		.04		0.296^a^		0.001^a^	
	Mean heart rate (bpm)	0.599 (1, 126)		.44		–0.046		0.604	
	Mean SDNN (ms)	0.133 (1, 126)		.72		0.098		0.274	
	Mean RMSSD (ms)	0.010 (1, 126)		.92		0.106		0.237	
	Relaxation	0.843 (1, 126)		.36		0.154		0.083	
	Relaxation stability	0.015 (1, 126)		.90		–0.182^a^		0.041	
	Attention	5.698 (1, 126)		.02		0.126		0.160	
	Attention stability	4.666 (1, 126)		.03		0.167		0.060	
**Negative affect**									
	Breathing rate (bpm)	0.061 (1, 126)		.81		0.177^a^		0.046	
	Mean heart rate (bpm)	6.880 (1, 126)		.01^a^		–0.133		0.137	
	Mean SDNN (ms)	1.704 (1, 126)		.19		0.106		0.237	
	Mean RMSSD (ms)	4.809 (1, 126)		.03		0.225^a^		0.011	
	Relaxation	0.060 (1, 126)		.81		0.103		0.247	
	Relaxation stability	1.510 (1, 126)		.22		–0.229^a^		0.010^a^	
	Attention	0.569 (1, 126)		.45		–0.062		0.489	
	Attention stability	3.928 (1, 126)		.05		–0.087		0.333	

^a^*P*<.0125.

^b^SDNN: standard deviation of normal to normal interval.

^c^RMSSD: root mean square of successive differences.

We compared the 7 dimensions of game experience to assess their impact on cognitive load, followed by partial correlation analysis. The results indicate that competence, immersion, challenge, and positive affect significantly influenced overall cognitive load. The specific effects of each dimension are detailed below.

Competence significantly impacted mental load (*F*_1,131_=12.639, *P*=.001) with a notable negative correlation (*r*=–0.301, *P*<.001). In addition, competence significantly influenced performance (*F*_1,131_=9.902, *P*=.002) and frustration (*F*_1,131_=10.168, *P*=.002), both with negative correlations (*r*=–0.542, *P*<.001) and (*r*=–0.554, *P*<.001), respectively.

Immersion affected mental load (*F*_1,131_=9.523, *P*=.003) with a significant negative correlation (*r*=–0.240, *P*=.006).

Challenge significantly influenced effort (*F*_1,131_=17.471, *P*<.001) and frustration (*F*_1,131_=8.012, *P*=.005) both exhibiting significant positive correlations (*r*=0.441, *P*<.001), and (*r*=0.586, *P*<.001).

Positive affect influenced mental load (*F*_1,131_=7.264, *P*=.008); however, the partial correlations were not significant, indicating a weaker direct impact under different interface conditions.

### Impact of Cognitive Load on Relaxation

We compared the 6 dimensions of cognitive load on relaxation, with MANOVA and partial correlation analysis results presented in Table 4.

MANOVA results indicate that mental demand and temporal demand were the primary factors influencing relaxation. Mental demand significantly affected attention (*F*_1,126_=13.395, *P*<.001), with a strong negative correlation (*r*=–0.308, *P*<.001); in other words, higher mental demand corresponds to lower attention. Temporal demand significantly impacted breathing rate (*F*_1,126_=7.900, *P*=.006), with a significant positive correlation (*r*=0.245, *P*=.005); thus, higher temporal demand increases breathing rate.

### Impact of Gaming Experience on Relaxation

Results from MANOVA and partial correlation analyses are shown in Table 5.

The study compared whether game experience influences relaxation. The statistical results indicate that flow, tension, and negative affect were the main gaming elements affecting overall relaxation. Flow significantly affected mean heart rate (*F*_1,126_=7.926, *P*=.006), mean SDNN (*F*_1,126_=8.132, *P*=.005), mean RMSSD (*F*_1,126_=11.171, *P*=.001), and attention (*F*_1,126_=7.420, *P*=.007). However, partial correlation analysis revealed no significant correlations between flow and these 4 variables, indicating a weak direct impact after controlling for different interface variables.

Tension significantly affected mean heart rate (*F*_1,126_=6.938, *P*=.010); however, there was no significant correlation. Negative affect significantly affected mean heart rate (*F*_1,126_=6.880, *P*=.01); however, there was no significant correlation.

### Correlation Between Cognitive Load and Insomnia Severity

**Figure 5:**
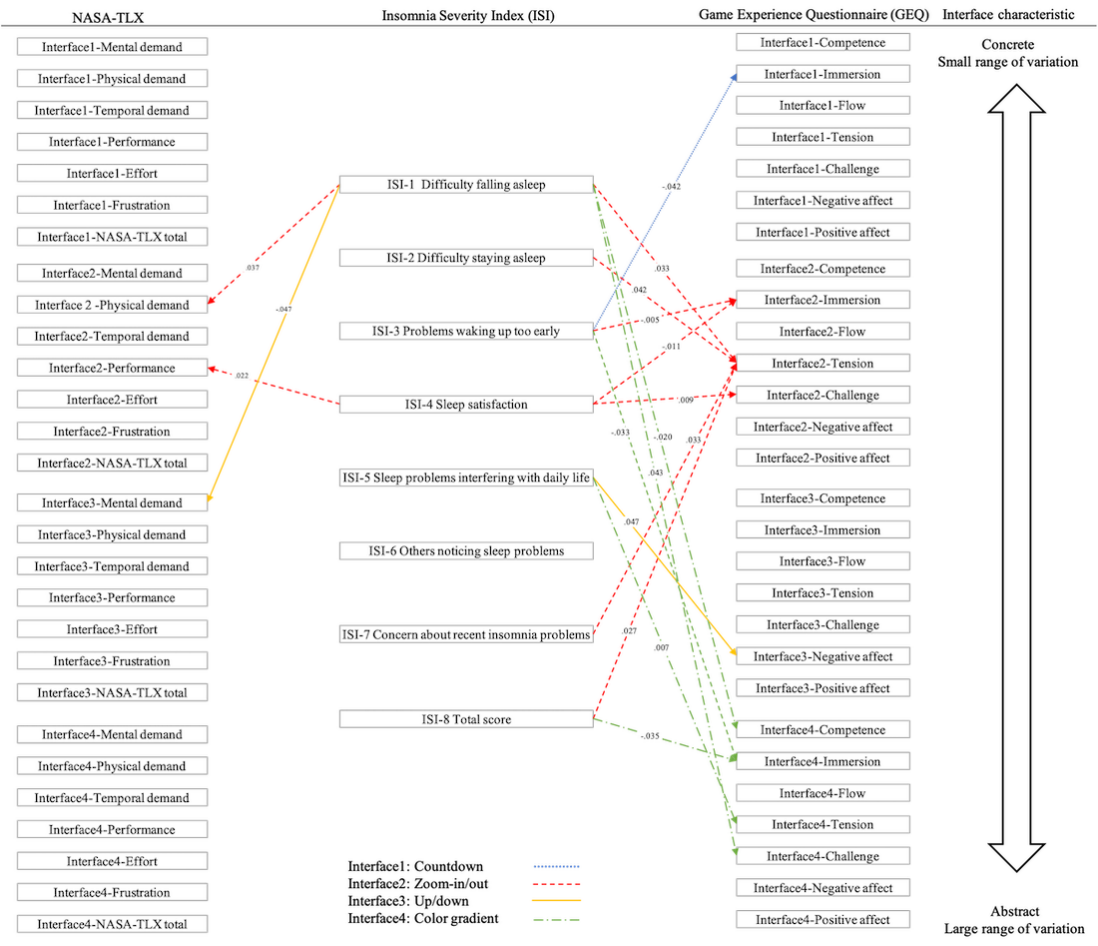
Correlations among insomnia severity, cognitive load, and gaming experience for 4 interfaces (countdown, zoom-in/out, up/down, and color gradients). NASA-TLX: NASA Task Load Index.

As shown in Figure 5, the correlation between cognitive load and insomnia severity was low. “Difficulty falling asleep” (ISI-1) was positively correlated with the physical demand of the zoom-in/out interface (*r*=0.364, *P*=.04) and negatively correlated with the mental demand of the up/down interface (*r*=–0.348, *P*=.047).

For the zoom-in/out interface, there was a positive correlation between performance and “sleep satisfaction” (ISI-4; *r*=0.399, *P*=.02).

There was a negative correlation between participants who had problems waking up too early (ISI-3) and immersion when using the countdown (*r*=–0.356, *P*=.04), zoom-in/out (*r*=–0.481, *P*=.005), and color gradient (*r*=–0.373, *P*=.03) interfaces, suggesting that immersion is a design element that can significantly impact insomnia severity. The zoom-in/out interface showed a moderate positive correlation between tension and “difficulty falling asleep” (ISI-1; *r*=0.372, *P*=.03), “difficulty staying asleep” (ISI-2; *r*=0.357, *P*=.04), “concerns about recent insomnia problems” (ISI-7; *r*=0.372, *P*=.03), and the total ISI score (*r*=0.385, *P*=.03).

The negative affect triggered by the up/down interface was positively correlated with “sleep problems interfering with daily life” (ISI-5; *r*=0.348, *P*=.047). For the color gradient interface, there was a positive correlation between tension and “sleep problems interfering with daily life” (ISI-5; *r*=0.464, *P*=.007).

Overall, the gaming experiences of the zoom-in/out and color gradient interfaces show greater correlations with insomnia severity.

### Sleep Self-Efficacy Correlation Analysis

**Figure 6:**
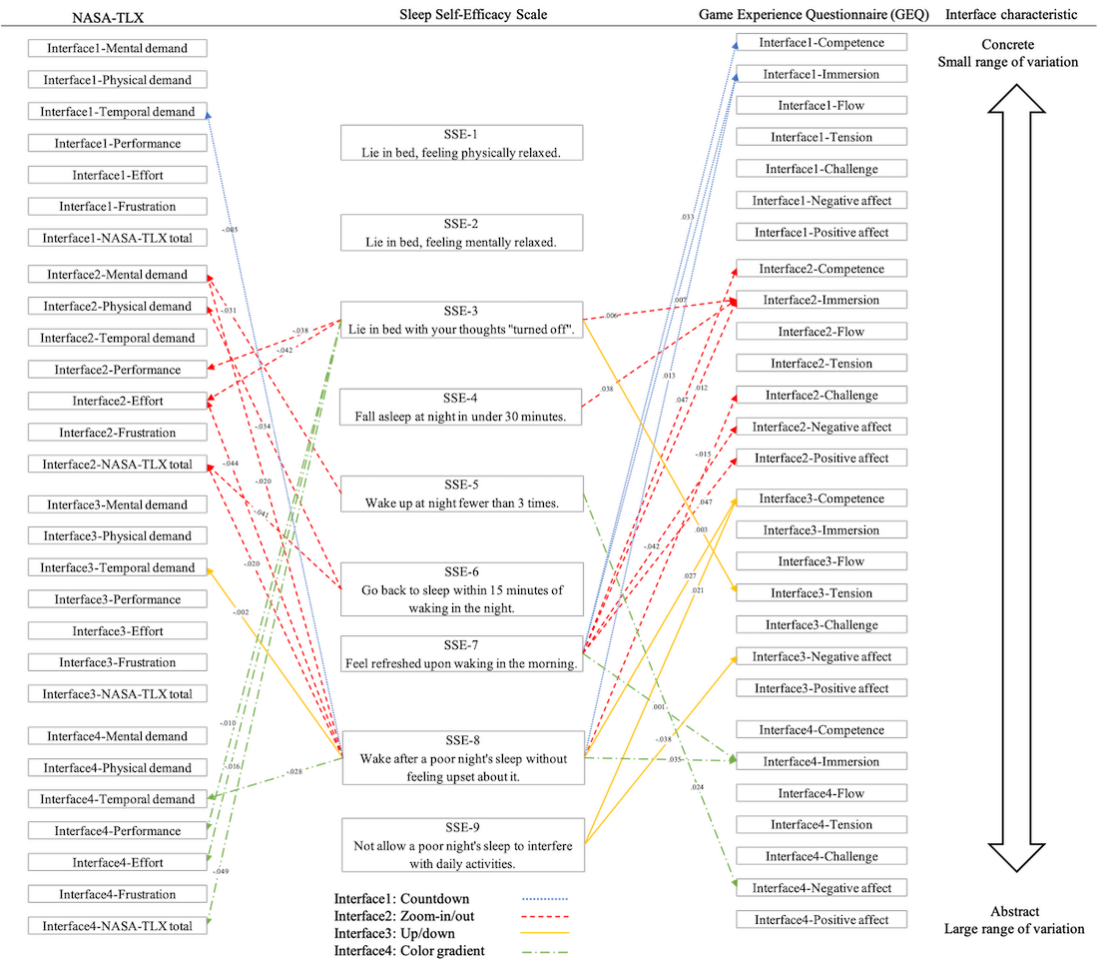
Correlations among sleep self-efficacy, cognitive load, and gaming experience for 4 interfaces (countdown, zoom-in/out, up/down, and color gradients). NASA-TLX: NASA Task Load Index; SSE: sleep self-efficacy.

As shown in Figure 6, the temporal demand of the countdown (*r*=–0.474, *P*=.005), up/down (*r*=–0.512, *P*=.002), and color gradient (*r*=–0.381, *P*=.03) interfaces were significantly negatively correlated with “wake after a poor night’s sleep without feeling upset about it” (SSE-8 [sleep self-efficacy]). This suggests that temporal demand is a factor that easily affects sleep self-efficacy.

The cognitive load of the color gradient interface demonstrated several significant negative correlations with sleep self-efficacy (SSE-3), including performance (*r*=–0.442, *P*=.01), effort (*r*=–0.415, *P*=.02), and NASA-TLX total (*r*=–0.345, *P*=.049).

Overall, the cognitive load of the zoom-in/out interface was negatively correlated with many aspects of sleep self-efficacy. “Lie in bed with thoughts turned off” (SSE-3) showed significant negative correlations with performance (*r*=–0.364, *P*=.04) and effort (*r*=–0.356, *P*=.04). “Go back to sleep within 15 minutes of waking in the night” (SSE-6) demonstrated significant negative correlations with physical demand (*r*=–0.370, *P*=.03) and NASA-TLX total (*r*=–0.358, *P*=.04). “Wake after a poor night’s sleep without feeling upset about it” (SSE-8) was significantly negatively correlated with mental demand (*r*=–0.402, *P*=.02), effort (*r*=–0.353, *P*=.04), and NASA-TLX total (*r*=–0.403, *P*=.02).

There were many correlations between the 7 dimensions of the GEQ and sleep self-efficacy, especially with the items “feel refreshed upon waking in the morning” (SSE-7) and “wake after a poor night’s sleep without feeling upset about it” (SSE-8), indicating that there is a relationship between game design elements and mental states upon waking. Competence and immersion were the 2 game dimensions most related to sleep self-efficacy. “Feel refreshed upon waking in the morning” (SSE-7) was positively correlated with competence (*r*=0.372, *P*=.03) and immersion (*r*=0.458, *P*=.007) for the countdown interface, competence (*r*=0.348, *P*=.047) and immersion (*r*=0.434, *P*=.01) for the zoom-in/out interfaces, and immersion (*r*=0.567, *P*=.001) for the color gradient interface. “Waking after a poor night’s sleep without feeling upset about it” (SSE-8) was positively correlated with immersion for the countdown interface (*r*=0.427, *P*=.01), competence (*r*=0.384, *P*=.03) for the up/down interface, and immersion (*r*=0.368, *P*=.04) for the color gradient interface. This indicates that higher competence or immersion in these interfaces correlated with higher self-efficacy upon waking up.

Negative affect also has an important impact on sleep self-efficacy. There was a significant positive correlation between the negative affect triggered by the color gradient interface and “wake up at night fewer than 3 times” (SSE-5; *r*=0.393, *P*=.02), a negative correlation between negative affect triggered by the zoom-in/out interface and “feel refreshed upon waking in the morning” (SSE-7; *r*=–0.356, *P*=.04), and a negative correlation between negative affect triggered by the up/down interface and “wake after a poor night’s sleep without feeling upset about it” (SSE-8; *r*=–0.363, *P*=.04). This indicates that negative affect can have both positive and negative impacts on sleep self-efficacy.

### Physiological and Psychological Values

**Figure 7:**
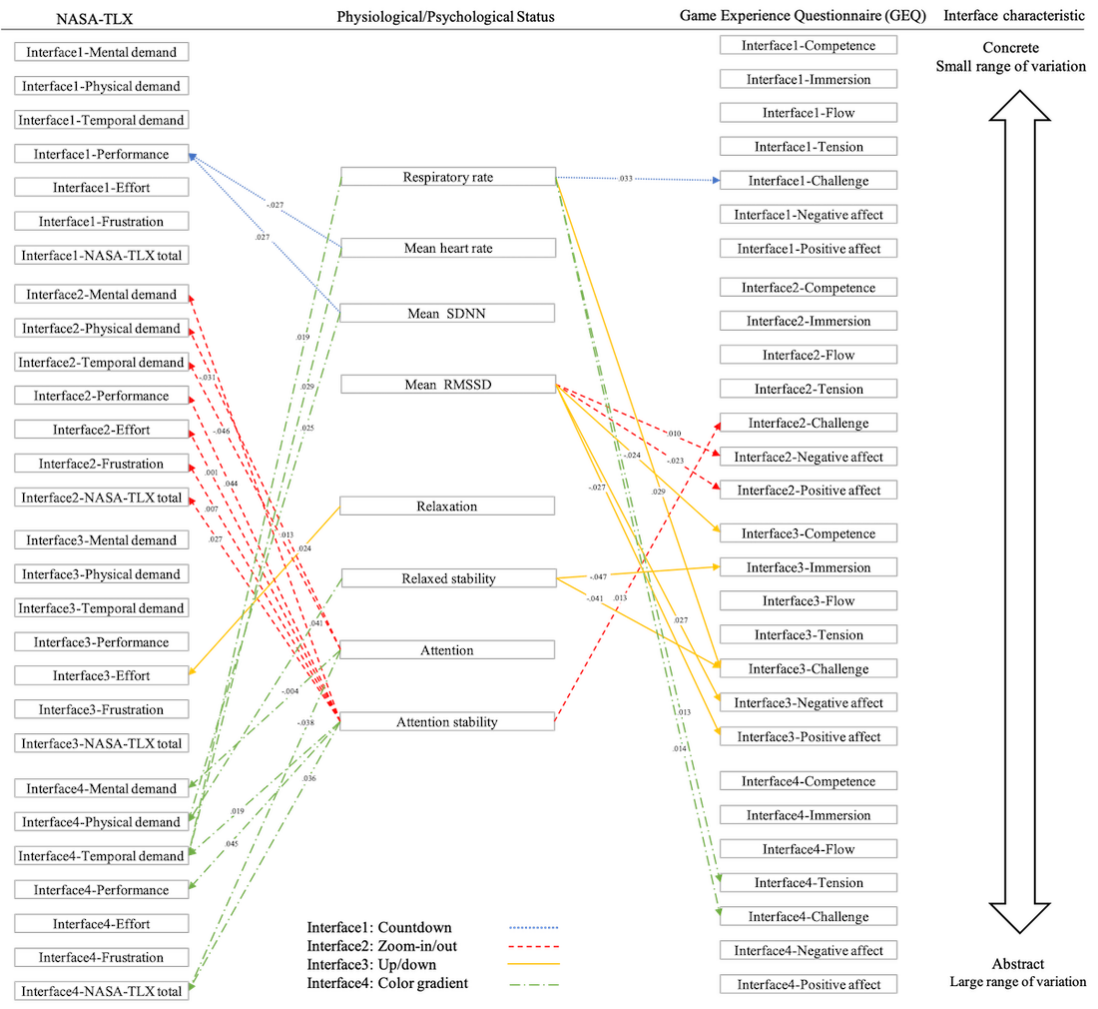
Correlations among physiological/psychological signals, cognitive load, and gaming experience for 4 interfaces (countdown, zoom-in/out, up/down, and color gradients). NASA-TLX: NASA Task Load Index; RMSSD: root mean square of successive differences; SDNN: standard deviation of normal to normal interval.

As shown in Figure 7, the countdown guidance mechanism had the most obvious effect on physiological states, particularly subjective performance as well as mean heart rate (*r*=–0.385, *P*=.03) and mean SDNN (*r*=0.385, *P*=.03). For the zoom-in/out interface, attention was negatively correlated with mental demand (*r*=–0.377, *P*=.03) and temporal demand (*r*=0.350, *P*=.046). Additionally, attention stability exhibited positive correlations with mental load (*r*=0.427, *P*=.01), effort (*r*=0.539, *P*=.001), frustration (*r*=0.458, *P*=.007), and overall score (*r*=0.385, *P*=.03). The limited range and specific guidance of this interface likely influenced attention. The effort and relaxation associated with the up/down interface were positively correlated (*r*=0.392, *P*=.02).

The color gradient interface was correlated with both physiological and psychological states, particularly in relation to temporal demand. Temporal demand was positively correlated with respiratory rate (*r*=0.413, *P*=.02), mean heart rate (*r*=0.380, *P*=.03), and attention stability (*r*=0.405, *P*=.02). Conversely, attention was negatively correlated with mental demand (*r*=–0.493, *P*=.004) and NASA-TLX total (*r*=–0.362, *P*=.04). Physical demand showed positive correlations with mean SDNN (r=0.389, *P*=.02) and relaxed stability (*r*=0.358, *P*=.04). While NASA-TLX total was negatively correlated with attention (*r*=–0.362, *P*=.04), it exhibited a positive correlation with attention stability (*r*=0.367, *P*=.04).

The challenge aspect of the game experience was highly correlated with the breathing rate. The challenge aspects of the countdown (*r*=0.378, *P*=.03), up/down (*r*=0.387, *P*=.03), and color gradient (*r*=0.440, *P*=.01) interfaces were all positively correlated with the breathing rate.

The up/down interface showed the most correlations with physiological/psychological signals. Relaxed stability was negatively correlated with immersion (*r*=–0.348, *P*=.047) and challenge (*r*=0.358, *P*=.04). Additionally, mean RMSSD was negatively correlated with competence (*r*=–0.391, *P*=.02).

The most significant game experiences affecting average RMSSD were positive and negative affect. The average RMSSD of the zoom-in/out interface was negatively correlated with positive affect (*r*=–0.395, *P*=.02) and positively correlated with negative affect (*r*=0.442, *P*=.01). The average RMSSD during the up/down interface was also negatively correlated with positive affect (*r*=–0.385, *P*=.03) and positively correlated with negative affect (*r*=0.384, *P*=.03).

### Qualitative Findings and Implications

The study compiled interview results from 33 participants. Users felt that the countdown interface provided clear guidance. However, the rapid change of numbers disrupted their breathing rhythm, and the prolonged inhalation and exhalation periods made it difficult to follow the guide. The zoom-in/out guidance mechanism is common in many apps, making it familiar and easy for users to understand and accept. However, care must be taken with the scale of the visual changes and the brightness of the colors, as excessive magnification or overly bright colors can cause visual strain. The up/down guidance, which mimics the natural rhythm of ocean waves, is visually soothing and facilitates relaxation, making it the preferred choice for most participants. When paired with music, it creates a sense of immersion, though the sensation of rising might cause some users to feel overwhelmed; therefore, it is crucial to consider the range of visual changes in the design. Users noted that the zoom-in/out and up/down guidance mechanisms align well with the body’s natural breathing patterns, enhancing comprehension and effectiveness. This finding supports that of Chuanromanee and Metoyer [43]. The color gradients, which are a more abstract guidance method, offered a high tolerance for error, which some users liked as it allowed them to match their own breathing rate, resulting in less pressure. However, the lack of clarity in the variations when first using this interface might disrupt the breathing rhythm, and the brightening colors could cause visual stimulation.

The effectiveness of different interfaces varies with individual differences. The interviews revealed that even with the same breathing rate, different guidance mechanisms gave users different perceptions of the rate, as visual animations, colors, and changes in size also affected subjective pressure and relaxation effects. Most participants felt that the initial stages were visually guided; however, after prolonged use, they shifted to auditory guidance. Therefore, visual and auditory guidance should complement each other, allowing users to become familiar with the breathing rate and subsequently continue with auditory guidance. Future app designs should incorporate more personalized options, such as selecting the breathing guidance rate, as many participants reported difficulty keeping up with the 6 breaths per minute initially. Enhancing auditory and visual changes, like voice and other animation guidance, should also be considered. Additionally, introducing stories or reward mechanisms could enrich the overall app experience and enhance user compliance with relaxation training.

## Discussion

### Principal Findings

#### Overview

In our 2-stage experiment, the first stage revealed that the zoom-in/out interface demanded the lowest cognitive load and achieved the highest user experience score, while the color gradient interface demanded the highest cognitive load. However, in the second stage, the color gradient interface was reported to demand the lowest overall cognitive load, followed by the up/down interface, which was also associated with the lowest breathing rate and mean heart rate as well as the highest mean RMSSD. This indicates that the duration of the diaphragmatic breathing game affects cognitive load and relaxation. For short-term use, the zoom-in/out interface incurs a lower cognitive load due to its moderate variation range and specific guidance, making it more effective initially. However, for longer durations, the more abstract up/down and color gradient interfaces are more effective, as they allow users to breathe at their own pace, reducing cognitive load and enhancing relaxation.

The countdown had high cognitive load in both stages and low relaxation in the second stage, indicating that specific numerical guidance causes higher stress regardless of duration, leading to poor relaxation outcomes. According to the results, abstract guidance generated a lower cognitive load, aligning with our hypothesis. However, abstract guidance demonstrated a more significant effect on influencing breathing rate compared with concrete guidance, contradicting our hypothesis.

#### Countdown

This is the most specific guidance with the smallest range of variation, primarily related to physiological relaxation. Subjective performance with this interface is significantly correlated with mean heart rate and SDNN. Additionally, participants with lower sleep self-efficacy experienced higher temporal demands, which impacted relaxation. The immersion experience with this interface is negatively correlated with users troubled by waking too early. Therefore, the applicability of countdown guidance in relaxation training games should be carefully considered.

#### Zoom-In/Out

This interface strikes a balance between abstract and specific guidance with a smaller range of variation. It shows more positive correlations between cognitive load and insomnia severity. There is also a significant negative correlation between cognitive load and sleep self-efficacy, making it unsuitable for users with sleep problems. Cognitive load with this interface is significantly correlated with attention and attention stability, requiring higher attention stability to complete tasks, thus increasing overall cognitive load.

#### Up/Down

This abstract interface with a larger range of variation shows no significant correlation between cognitive load and insomnia severity, sleep self-efficacy, or physiological and psychological relaxation, indicating that it is less influenced by differences in user groups and is thus suitable for most users. There is a negative correlation between difficulty falling asleep and mental demand for this interface, which indicates that it is more effective for those with sleep onset difficulties. The negative affect triggered by this interface is positively correlated with mean RMSSD.

#### Color Gradients

This interface is the most abstract with the largest range of variation. There is a significant negative correlation between cognitive load and the sleep self-efficacy item “lie in bed with your thoughts turned off” (SSE-3), indicating that abstract and highly variable visual guidance requires more effort to understand and adapt to. Additionally, the cognitive load of this interface shows significant correlations with breathing rate, attention, and attention stability, particularly with a positive correlation between temporal demand and breathing rate. The abstract nature of the guidance increases temporal pressure and breathing rate. At the same time, understanding the abstract changes demands sustained focus, affecting both attention and cognitive load.

In terms of game experience, a sense of competence, immersion, challenge, and positive affect influence cognitive load, particularly mental and temporal load, subjective performance, and frustration. Higher competence and immersion reduce subjective cognitive load. However, challenges are associated with increased effort and frustration. Therefore, relaxation training should enhance competence and immersion while reducing challenges to lower cognitive load. Furthermore, sleep self-efficacy is also strongly correlated with competence, immersion, and temporal demand. Therefore, attention should be given to the stress caused by the time requirements of the guidance mechanisms.

Mental and temporal demands significantly impact attention and breathing rate. Higher mental load decreases attention, while higher temporal demand increases breathing rate. Therefore, breathing guidance interfaces should minimize time pressure. Game-induced flow, tension, and negative affects impact various physiological and psychological relaxation indicators.

The correlation analysis revealed that the challenge was positively associated with breathing rate in multiple instances. Additionally, negative affect was significantly positively correlated with mean RMSSD. Because negative affects such as boredom or weariness also indicate lower stimulation in a low-stimulation environment, people tend to feel more relaxed. Conversely, positive affect includes fun, enjoyment, or pleasure, which may shift attention to the game interface rather than the act of diaphragmatic breathing itself, leading to reduced physiological relaxation. However, emotional responses themselves are quite complex, so future research could delve deeper into their impact on relaxation.

### Limitations

There are several limitations to our study. First, it focused on analyzing 4 specific guidance mechanisms and considered only the dependent variables of cognitive load, game experience, relaxation, and attention, without including a direct comparison to traditional video-based teaching. The experiments were not restricted to being conducted exclusively during the day or prior to sleep, so the therapeutic effects on insomnia were not within the scope of this study. In future studies, diaphragmatic breathing games could be incorporated into brief behavioral treatments for insomnia, with experiments conducted closer to bedtime. Through long-term observation and follow-up, it would be possible to determine whether gamified relaxation training can enhance the effectiveness of insomnia treatment.

Another limitation of the study was the difficulty in recruiting insomniacs during the implementation of the study, resulting in a limited number of participants with severe insomnia. Future studies are recommended to include a larger sample of insomniacs to improve the generalizability and statistical power of the findings.

Finally, In phase 1 of the study, the order of the guidance mechanisms was not counterbalanced, and all participants followed the same sequence. Future studies should consider using counterbalancing techniques to control for potential order effects.

### Conclusions

In the design of diaphragmatic breathing guidance for patients with insomnia, the study results indicate that the up/down interface is the most effective, as it results in lower cognitive load and higher relaxation. Both gaming experience and cognitive load significantly affect relaxation, with psychological and temporal demands having a notable impact on breathing rate. Positive and negative affect are critical factors influencing relaxation, with negative affect associated with higher relaxation effects. These findings offer valuable insights into the design of diaphragmatic breathing training, suggesting that appropriate guidance methods and gamified designs can enhance relaxation effects. Recommendations are made for the design of presleep breathing guidance and relaxation training apps.

## Appendix

Multimedia Appendix 1 Correlations among variables for four interfaces.

## Abbreviations

BBTI: brief behavioral treatment for insomnia
CBT-I: cognitive behavioral therapy for insomnia
EEG: electroencephalogram
GEQ: Game Experience Questionnaire
HQ-I: hedonic quality-identity
HQ-S: hedonic quality-stimulation
HRV: heart rate variability
ISI: Insomnia Severity Index
NASA-TLX: NASA Task Load Index
PQ: pragmatic quality
RMSSD: root mean square of successive differences
SDNN: standard deviation of normal to normal interval
SSE: sleep self-efficacy
