# Supplementary material for: Diaphragmatic Breathing Interfaces to Promote Relaxation for Mitigating Insomnia: Pilot Study
Source: JMIR Serious Games. 2025 Mar 4;13:e67000. doi: 10.2196/67000 (PMC11920670; doi:10.2196/67000)
Supplement: Multimedia Appendix 1 [file games_v13i1e67000_app1.docx]

1. **Correlations among insomnia severity and cognitive load for four interfaces.**

Interface1 (Countdown)

| Variable | ISI-1 Difficulty falling asleep | ISI-2 Difficulty staying asleep | ISI-3 Problems waking up too early | ISI-4 Sleep satisfaction | ISI-5 Sleep problems interfering with daily life | ISI-6 Others noticing sleep problems | ISI-7 Concern about recent insomnia problems | ISI-8 Total Score |
| --- | --- | --- | --- | --- | --- | --- | --- | --- |
| Mental demand | | | | | | | | |
| r | -.019 | -.083 | .087 | .249 | .056 | -.002 | .044 | .064 |
| P-value  (two-tailed test) | .918 | .646 | .632 | .163 | .755 | .993 | .808 | .725 |
| Physical demand | | | | | | | | |
| r | .309 | .078 | .128 | .214 | .270 | .142 | .114 | .231 |
| P-value  (two-tailed test) | .080 | .668 | .477 | .233 | .129 | .431 | .527 | .196 |
| Temporal demand | | | | | | | | |
| r | .092 | .154 | .046 | .223 | .063 | .062 | .086 | .130 |
| P-value  (two-tailed test) | .609 | .391 | .798 | .212 | .728 | .731 | .636 | .471 |
| Performance | | | | | | | | |
| r | -.165 | -.109 | -.197 | .026 | -.090 | -.021 | -.250 | -.147 |
| P-value  (two-tailed test) | .357 | .547 | .271 | .884 | .618 | .909 | .160 | .415 |
| Effort | | | | | | | | |
| r | -.004 | .015 | -.048 | .140 | .010 | .004 | -.012 | .018 |
| P-value  (two-tailed test) | .981 | .935 | .789 | .437 | .956 | .983 | .946 | .919 |
| Frustration | | | | | | | | |
| r | -.256 | .006 | -.166 | -.050 | -.043 | .008 | -.246 | -.136 |
| P-value  (two-tailed test) | .151 | .972 | .356 | .783 | .813 | .963 | .167 | .450 |
| NASA-TLX total | | | | | | | | |
| r | .007 | .032 | -.026 | .199 | .076 | .055 | -.050 | .054 |
| P-value  (two-tailed test) | .969 | .861 | .885 | .266 | .675 | .762 | .781 | .767 |

Interface2 (Zoom in/out)

| Variable | ISI-1 Difficulty falling asleep | ISI-2 Difficulty staying asleep | ISI-3 Problems waking up too early | ISI-4 Sleep satisfaction | ISI-5 Sleep problems interfering with daily life | ISI-6 Others noticing sleep problems | ISI-7 Concern about recent insomnia problems | ISI-8 Total Score |
| --- | --- | --- | --- | --- | --- | --- | --- | --- |
| Mental demand | | | | | | | | |
| r | .131 | -.044 | -.022 | .303 | .104 | -.033 | .202 | .119 |
| P-value  (two-tailed test) | .469 | .808 | .904 | .086 | .565 | .854 | .261 | .510 |
| Physical demand | | | | | | | | |
| r | .364 | .255 | .175 | .301 | .247 | .302 | .216 | .338 |
| P-value  (two-tailed test) | .037 | .152 | .331 | .089 | .166 | .088 | .228 | .054 |
| Temporal demand | | | | | | | | |
| r | -.036 | -.147 | -.288 | -.041 | .120 | -.065 | -.017 | -.081 |
| P-value  (two-tailed test) | .840 | .415 | .104 | .821 | .505 | .717 | .927 | .654 |
| Performance | | | | | | | | |
| r | -.056 | .008 | -.187 | -.028 | -.059 | -.177 | -.147 | -.122 |
| P-value  (two-tailed test) | .758 | .966 | .296 | .879 | .744 | .324 | .414 | .501 |
| Effort | | | | | | | | |
| r | .248 | .234 | .119 | .399 | .099 | .019 | .222 | .239 |
| P-value  (two-tailed test) | .165 | .190 | .511 | .022 | .585 | .917 | .214 | .181 |
| Frustration | | | | | | | | |
| r | .236 | .340 | .171 | .303 | .129 | .052 | .149 | .244 |
| P-value  (two-tailed test) | .187 | .053 | .342 | .086 | .476 | .776 | .407 | .171 |
| NASA-TLX total | | | | | | | | |
| r | .204 | .148 | -.001 | .278 | .148 | .032 | .144 | .172 |
| P-value  (two-tailed test) | .256 | .410 | .994 | .118 | .410 | .861 | .424 | .340 |

Interface3 (Up /down)

| Variable | ISI-1 Difficulty falling asleep | ISI-2 Difficulty staying asleep | ISI-3 Problems waking up too early | ISI-4 Sleep satisfaction | ISI-5 Sleep problems interfering with daily life | ISI-6 Others noticing sleep problems | ISI-7 Concern about recent insomnia problems | ISI-8 Total Score |
| --- | --- | --- | --- | --- | --- | --- | --- | --- |
| Mental demand | | | | | | | | |
| r | -.348 | -.046 | -.158 | .047 | -.219 | -.118 | -.052 | -.165 |
| P-value  (two-tailed test) | .047 | .797 | .379 | .794 | .220 | .513 | .776 | .359 |
| Physical demand | | | | | | | | |
| r | .140 | .143 | .040 | .243 | .054 | .079 | .079 | .139 |
| P-value  (two-tailed test) | .438 | .428 | .825 | .173 | .765 | .664 | .662 | .441 |
| Temporal demand | | | | | | | | |
| r | -.036 | .025 | -.204 | .176 | -.086 | -.172 | .027 | -.053 |
| P-value  (two-tailed test) | .841 | .892 | .254 | .326 | .633 | .339 | .880 | .768 |
| Performance | | | | | | | | |
| r | .003 | -.040 | -.263 | -.067 | -.025 | .046 | -.088 | -.078 |
| P-value  (two-tailed test) | .985 | .827 | .139 | .710 | .890 | .798 | .627 | .664 |
| Effort | | | | | | | | |
| r | .101 | .157 | .025 | .213 | .007 | -.031 | .024 | .085 |
| P-value  (two-tailed test) | .577 | .384 | .889 | .235 | .971 | .865 | .893 | .638 |
| Frustration | | | | | | | | |
| r | -.084 | .025 | -.235 | -.125 | .120 | .216 | -.116 | -.032 |
| P-value  (two-tailed test) | .644 | .892 | .187 | .488 | .506 | .227 | .519 | .858 |
| NASA-TLX total | | | | | | | | |
| r | -.041 | .064 | -.157 | .126 | -.037 | -.008 | -.016 | -.015 |
| P-value  (two-tailed test) | .819 | .724 | .383 | .484 | .838 | .967 | .927 | .933 |

Interface4 (Color gradients)

| Variable | ISI-1 Difficulty falling asleep | ISI-2 Difficulty staying asleep | ISI-3 Problems waking up too early | ISI-4 Sleep satisfaction | ISI-5 Sleep problems interfering with daily life | ISI-6 Others noticing sleep problems | ISI-7 Concern about recent insomnia problems | ISI-8 Total Score |
| --- | --- | --- | --- | --- | --- | --- | --- | --- |
| Mental demand | | | | | | | | |
| r | -.011 | -.195 | -.242 | -.149 | .111 | .034 | -.168 | -.107 |
| P-value  (two-tailed test) | .950 | .276 | .175 | .409 | .540 | .852 | .349 | .555 |
| Physical demand | | | | | | | | |
| r | .202 | .050 | -.038 | .177 | .094 | .052 | .070 | .110 |
| P-value  (two-tailed test) | .259 | .780 | .832 | .323 | .602 | .774 | .699 | .542 |
| Temporal demand | | | | | | | | |
| r | .210 | .294 | -.050 | .004 | .236 | .139 | .099 | .168 |
| P-value  (two-tailed test) | .240 | .096 | .783 | .981 | .187 | .439 | .585 | .351 |
| Performance | | | | | | | | |
| r | .062 | -.042 | -.240 | .064 | .015 | -.079 | -.108 | -.061 |
| P-value  (two-tailed test) | .730 | .815 | .178 | .725 | .935 | .661 | .550 | .736 |
| Effort | | | | | | | | |
| r | .257 | .081 | .004 | .256 | .135 | -.035 | .137 | .151 |
| P-value  (two-tailed test) | .148 | .652 | .982 | .150 | .454 | .845 | .446 | .403 |
| Frustration | | | | | | | | |
| r | .153 | .043 | -.199 | .102 | .108 | .055 | .006 | .049 |
| P-value  (two-tailed test) | .394 | .813 | .266 | .573 | .551 | .763 | .974 | .788 |
| NASA-TLX total | | | | | | | | |
| r | .209 | .073 | -.164 | .102 | .171 | .048 | .020 | .083 |
| P-value  (two-tailed test) | .244 | .686 | .360 | .571 | .342 | .791 | .913 | .644 |

1. **Correlations among insomnia severity and gaming experience for four interfaces.**

Interface1 (Countdown)

| Variable | ISI-1 Difficulty falling asleep | ISI-2 Difficulty staying asleep | ISI-3 Problems waking up too early | ISI-4 Sleep satisfaction | ISI-5 Sleep problems interfering with daily life | ISI-6 Others noticing sleep problems | ISI-7 Concern about recent insomnia problems | ISI-8 Total Score |
| --- | --- | --- | --- | --- | --- | --- | --- | --- |
| Competence | | | | | | | | |
| r | -.064 | -.148 | -.021 | -.145 | -.088 | -.194 | -.026 | -.124 |
| P-value  (two-tailed test) | .724 | .412 | .909 | .422 | .626 | .279 | .888 | .491 |
| Immersion | | | | | | | | |
| r | -.171 | -.308 | -.356 | -.290 | -.109 | -.181 | -.202 | -.290 |
| P-value  (two-tailed test) | .340 | .081 | .042 | .102 | .547 | .312 | .260 | .101 |
| Flow | | | | | | | | |
| r | -.147 | -.102 | .060 | -.029 | -.107 | -.075 | .113 | -.050 |
| P-value  (two-tailed test) | .416 | .573 | .738 | .875 | .552 | .679 | .532 | .781 |
| Tension | | | | | | | | |
| r | .204 | .186 | .070 | .191 | .299 | .318 | .045 | .242 |
| P-value  (two-tailed test) | .256 | .300 | .697 | .287 | .091 | .072 | .802 | .175 |
| Challenge | | | | | | | | |
| r | .181 | .284 | .130 | .207 | .062 | .179 | .054 | .194 |
| P-value  (two-tailed test) | .314 | .110 | .472 | .248 | .733 | .319 | .766 | .279 |
| Negative affect | | | | | | | | |
| r | .159 | .191 | .069 | .109 | .118 | .190 | -.043 | .142 |
| P-value  (two-tailed test) | .376 | .288 | .704 | .545 | .514 | .290 | .811 | .430 |
| Positive affect | | | | | | | | |
| r | -.119 | -.264 | -.170 | -.163 | -.102 | -.287 | -.002 | -.199 |
| P-value  (two-tailed test) | .508 | .137 | .343 | .366 | .572 | .106 | .990 | .267 |

Interface2 (Zoom in/out)

| Variable | ISI-1 Difficulty falling asleep | ISI-2 Difficulty staying asleep | ISI-3 Problems waking up too early | ISI-4 Sleep satisfaction | ISI-5 Sleep problems interfering with daily life | ISI-6 Others noticing sleep problems | ISI-7 Concern about recent insomnia problems | ISI-8 Total Score |
| --- | --- | --- | --- | --- | --- | --- | --- | --- |
| Competence | | | | | | | | |
| r | -.017 | .113 | .122 | -.103 | -.045 | .018 | -.029 | .008 |
| P-value  (two-tailed test) | .923 | .530 | .499 | .570 | .805 | .920 | .874 | .966 |
| Immersion | | | | | | | | |
| r | -.288 | -.279 | -.481 | -.439 | -.048 | .008 | -.311 | -.327 |
| P-value  (two-tailed test) | .104 | .116 | .005 | .011 | .793 | .963 | .078 | .063 |
| Flow | | | | | | | | |
| r | -.248 | -.084 | -.035 | .015 | -.229 | -.004 | -.056 | -.118 |
| P-value  (two-tailed test) | .164 | .641 | .845 | .936 | .200 | .983 | .759 | .515 |
| Tension | | | | | | | | |
| r | .372 | .357 | .265 | .280 | .294 | .184 | .372 | .385 |
| P-value  (two-tailed test) | .033 | .042 | .136 | .114 | .097 | .305 | .033 | .027 |
| Challenge | | | | | | | | |
| r | .320 | .328 | .124 | .446 | .092 | .075 | .322 | .304 |
| P-value  (two-tailed test) | .070 | .062 | .493 | .009 | .609 | .678 | .068 | .086 |
| Negative affect | | | | | | | | |
| r | .135 | .000 | -.041 | -.007 | .147 | .048 | .100 | .072 |
| P-value  (two-tailed test) | .453 | 1.000 | .820 | .969 | .413 | .792 | .581 | .688 |
| Positive affect | | | | | | | | |
| r | -.140 | -.092 | -.133 | -.052 | .039 | .115 | -.037 | -.049 |
| P-value  (two-tailed test) | .436 | .610 | .460 | .774 | .829 | .524 | .838 | .784 |

Interface3 (Up/down)

| Variable | ISI-1 Difficulty falling asleep | ISI-2 Difficulty staying asleep | ISI-3 Problems waking up too early | ISI-4 Sleep satisfaction | ISI-5 Sleep problems interfering with daily life | ISI-6 Others noticing sleep problems | ISI-7 Concern about recent insomnia problems | ISI-8 Total Score |
| --- | --- | --- | --- | --- | --- | --- | --- | --- |
| Competence | | | | | | | | |
| r | -.104 | -.169 | .056 | -.229 | -.187 | -.235 | -.141 | -.185 |
| P-value  (two-tailed test) | .564 | .346 | .755 | .200 | .296 | .188 | .434 | .302 |
| Immersion | | | | | | | | |
| r | .059 | -.125 | .013 | -.042 | -.042 | -.126 | -.025 | -.052 |
| P-value  (two-tailed test) | .744 | .488 | .943 | .816 | .817 | .486 | .888 | .773 |
| Flow | | | | | | | | |
| r | -.012 | -.076 | .259 | .068 | -.108 | -.151 | .061 | .006 |
| P-value  (two-tailed test) | .945 | .673 | .145 | .707 | .551 | .403 | .735 | .973 |
| Tension | | | | | | | | |
| r | .043 | -.081 | -.260 | -.105 | .337 | .268 | .056 | .059 |
| P-value  (two-tailed test) | .811 | .656 | .143 | .561 | .055 | .131 | .757 | .745 |
| Challenge | | | | | | | | |
| r | .166 | .218 | -.094 | .189 | .155 | .160 | .124 | .165 |
| P-value  (two-tailed test) | .357 | .223 | .603 | .293 | .390 | .375 | .492 | .358 |
| Negative affect | | | | | | | | |
| r | -.005 | -.127 | -.234 | -.066 | .348 | .273 | -.035 | .041 |
| P-value  (two-tailed test) | .977 | .481 | .190 | .715 | .047 | .124 | .845 | .823 |
| Positive affect | | | | | | | | |
| r | .032 | -.043 | .169 | -.019 | -.196 | -.241 | -.012 | -.062 |
| P-value  (two-tailed test) | .859 | .813 | .347 | .915 | .275 | .176 | .948 | .732 |

Interface4 (Color gradients)

| Variable | ISI-1 Difficulty falling asleep | ISI-2 Difficulty staying asleep | ISI-3 Problems waking up too early | ISI-4 Sleep satisfaction | ISI-5 Sleep problems interfering with daily life | ISI-6 Others noticing sleep problems | ISI-7 Concern about recent insomnia problems | ISI-8 Total Score |
| --- | --- | --- | --- | --- | --- | --- | --- | --- |
| Competence | | | | | | | | |
| r | -.405 | -.264 | -.119 | -.152 | -.249 | -.343 | -.226 | -.320 |
| P-value  (two-tailed test) | .020 | .137 | .509 | .398 | .162 | .051 | .206 | .070 |
| Immersion | | | | | | | | |
| r | -.312 | -.311 | -.373 | -.312 | -.259 | -.292 | -.170 | -.368 |
| P-value  (two-tailed test) | .077 | .079 | .033 | .077 | .145 | .099 | .344 | .035 |
| Flow | | | | | | | | |
| r | -.175 | -.202 | .102 | .049 | -.052 | -.085 | .048 | -.053 |
| P-value  (two-tailed test) | .331 | .260 | .572 | .787 | .772 | .639 | .791 | .772 |
| Tension | | | | | | | | |
| r | .256 | .278 | .122 | .086 | .464 | .364 | .141 | .316 |
| P-value  (two-tailed test) | .151 | .117 | .497 | .633 | .007 | .037 | .432 | .073 |
| Challenge | | | | | | | | |
| r | .354 | .172 | .019 | .234 | .277 | .166 | .274 | .274 |
| P-value  (two-tailed test) | .043 | .339 | .916 | .191 | .118 | .355 | .124 | .123 |
| Negative affect | | | | | | | | |
| r | .131 | -.010 | -.110 | -.182 | .301 | .255 | -.048 | .069 |
| P-value  (two-tailed test) | .468 | .957 | .540 | .311 | .089 | .151 | .789 | .703 |
| Positive affect | | | | | | | | |
| r | -.290 | -.209 | -.142 | -.112 | -.207 | -.342 | -.167 | -.268 |
| P-value  (two-tailed test) | .102 | .242 | .432 | .535 | .248 | .051 | .354 | .131 |

1. **Correlations among sleep self-efficacy and** **cognitive load for four interfaces.**

Interface1 (Countdown)

| Variable | SSE-1  Lie in bed, feeling physically relaxed. | SSE-2  Lie in bed, feeling mentally relaxed. | SSE-3  Lie in bed with your thoughts "turned off". | SSE-4  Fall asleep at night in under 30 minutes. | SSE-5  Wake up at night fewer than 3 times. | SSE-6  Go back to sleep within 15 minutes of waking in the night. | SSE-7  Feel refreshed upon waking in the morning. | SSE-8  Wake after a poor night's sleep without feeling upset about it. | SSE-9  Not allow a poor night's sleep to interfere with daily activities. |
| --- | --- | --- | --- | --- | --- | --- | --- | --- | --- |
| Mental demand | | | | | | | | | |
| r | -.116 | -.146 | -.132 | -.094 | -.289 | .085 | -.126 | -.183 | -.220 |
| P-value  (two-tailed test) | .521 | .419 | .463 | .604 | .103 | .637 | .484 | .308 | .218 |
| Physical demand | | | | | | | | | |
| r | -.181 | -.213 | -.084 | -.039 | -.071 | -.200 | .028 | -.167 | -.018 |
| P-value  (two-tailed test) | .313 | .233 | .641 | .829 | .695 | .265 | .875 | .353 | .919 |
| Temporal demand | | | | | | | | | |
| r | -.257 | -.295 | -.254 | -.125 | -.166 | .121 | -.342 | -.474 | -.077 |
| P-value  (two-tailed test) | .149 | .096 | .154 | .488 | .355 | .504 | .051 | .005 | .669 |
| Performance | | | | | | | | | |
| r | .132 | .105 | -.170 | .280 | .065 | -.146 | -.134 | .025 | -.186 |
| P-value  (two-tailed test) | .463 | .561 | .344 | .115 | .720 | .419 | .457 | .891 | .301 |
| Effort | | | | | | | | | |
| r | -.083 | -.106 | -.059 | .080 | .040 | -.194 | -.099 | -.082 | -.097 |
| P-value  (two-tailed test) | .646 | .556 | .743 | .658 | .825 | .280 | .582 | .649 | .589 |
| Frustration | | | | | | | | | |
| r | .120 | .180 | -.138 | .300 | .133 | -.135 | -.074 | .118 | -.122 |
| P-value  (two-tailed test) | .506 | .317 | .442 | .090 | .460 | .454 | .682 | .514 | .497 |
| NASA-TLX total | | | | | | | | | |
| r | -.110 | -.132 | -.209 | .082 | -.074 | -.105 | -.189 | -.208 | -.164 |
| P-value  (two-tailed test) | .541 | .463 | .244 | .649 | .682 | .561 | .293 | .245 | .361 |

Interface2 (Zoom in/out)

| Variable | SSE-1  Lie in bed, feeling physically relaxed. | SSE-2  Lie in bed, feeling mentally relaxed. | SSE-3  Lie in bed with your thoughts "turned off". | SSE-4  Fall asleep at night in under 30 minutes. | SSE-5  Wake up at night fewer than 3 times. | SSE-6  Go back to sleep within 15 minutes of waking in the night. | SSE-7  Feel refreshed upon waking in the morning. | SSE-8  Wake after a poor night's sleep without feeling upset about it. | SSE-9  Not allow a poor night's sleep to interfere with daily activities. |
| --- | --- | --- | --- | --- | --- | --- | --- | --- | --- |
| Mental demand | | | | | | | | | |
| r | -.194 | -.303 | -.305 | -.113 | -.377 | -.224 | -.134 | -.402 | -.246 |
| P-value  (two-tailed test) | .279 | .086 | .084 | .530 | .031 | .210 | .456 | .020 | .168 |
| Physical demand | | | | | | | | | |
| r | -.308 | -.283 | -.288 | -.126 | -.122 | -.370 | -.073 | -.303 | -.340 |
| P-value  (two-tailed test) | .081 | .111 | .104 | .485 | .499 | .034 | .687 | .086 | .053 |
| Temporal demand | | | | | | | | | |
| r | .169 | .030 | -.150 | .094 | -.035 | -.132 | .058 | -.269 | -.292 |
| P-value  (two-tailed test) | .347 | .867 | .405 | .604 | .849 | .463 | .747 | .130 | .100 |
| Performance | | | | | | | | | |
| r | .296 | .053 | -.364 | .103 | -.122 | -.244 | -.116 | -.186 | -.230 |
| P-value  (two-tailed test) | .094 | .770 | .038 | .569 | .499 | .172 | .521 | .300 | .198 |
| Effort | | | | | | | | | |
| r | -.195 | -.251 | -.356 | -.004 | -.075 | -.340 | -.256 | -.353 | -.268 |
| P-value  (two-tailed test) | .276 | .160 | .042 | .984 | .679 | .053 | .151 | .044 | .131 |
| Frustration | | | | | | | | | |
| r | -.128 | -.129 | -.089 | .068 | -.213 | -.300 | -.231 | -.305 | -.232 |
| P-value  (two-tailed test) | .479 | .474 | .623 | .708 | .235 | .090 | .195 | .084 | .194 |
| NASA-TLX total | | | | | | | | | |
| r | -.089 | -.199 | -.338 | .002 | -.206 | -.358 | -.164 | -.403 | -.358 |
| P-value  (two-tailed test) | .621 | .268 | .054 | .990 | .251 | .041 | .363 | .020 | .041 |

Interface3 (Up/down)

| Variable | SSE-1  Lie in bed, feeling physically relaxed. | SSE-2  Lie in bed, feeling mentally relaxed. | SSE-3  Lie in bed with your thoughts "turned off". | SSE-4  Fall asleep at night in under 30 minutes. | SSE-5  Wake up at night fewer than 3 times. | SSE-6  Go back to sleep within 15 minutes of waking in the night. | SSE-7  Feel refreshed upon waking in the morning. | SSE-8  Wake after a poor night's sleep without feeling upset about it. | SSE-9  Not allow a poor night's sleep to interfere with daily activities. |
| --- | --- | --- | --- | --- | --- | --- | --- | --- | --- |
| Mental demand | | | | | | | | | |
| r | -.001 | -.090 | -.153 | -.057 | -.330 | .077 | -.065 | -.152 | -.136 |
| P-value  (two-tailed test) | .996 | .620 | .395 | .753 | .060 | .672 | .718 | .397 | .449 |
| Physical demand | | | | | | | | | |
| r | -.123 | -.171 | -.190 | -.094 | -.291 | -.152 | -.057 | -.225 | -.059 |
| P-value  (two-tailed test) | .495 | .342 | .289 | .603 | .101 | .399 | .754 | .208 | .744 |
| Temporal demand | | | | | | | | | |
| r | .073 | -.154 | -.181 | -.140 | -.290 | .061 | -.277 | -.512 | -.249 |
| P-value  (two-tailed test) | .685 | .393 | .313 | .437 | .102 | .736 | .118 | .002 | .162 |
| Performance | | | | | | | | | |
| r | .164 | -.126 | -.211 | -.010 | -.077 | -.178 | -.034 | -.110 | -.215 |
| P-value  (two-tailed test) | .361 | .484 | .238 | .954 | .670 | .321 | .853 | .543 | .229 |
| Effort | | | | | | | | | |
| r | .002 | -.051 | -.289 | -.140 | -.301 | -.213 | .023 | -.196 | -.132 |
| P-value  (two-tailed test) | .990 | .777 | .103 | .438 | .089 | .235 | .898 | .273 | .464 |
| Frustration | | | | | | | | | |
| r | .055 | -.012 | .022 | .167 | -.013 | -.203 | .020 | .040 | -.224 |
| P-value  (two-tailed test) | .760 | .949 | .905 | .353 | .942 | .258 | .914 | .827 | .210 |
| NASA-TLX total | | | | | | | | | |
| r | .027 | -.135 | -.225 | -.073 | -.298 | -.124 | -.091 | -.268 | -.213 |
| P-value  (two-tailed test) | .879 | .455 | .209 | .686 | .092 | .493 | .615 | .132 | .235 |

Interface4 (Color gradients)

| Variable | SSE-1  Lie in bed, feeling physically relaxed. | SSE-2  Lie in bed, feeling mentally relaxed. | SSE-3  Lie in bed with your thoughts "turned off". | SSE-4  Fall asleep at night in under 30 minutes. | SSE-5  Wake up at night fewer than 3 times. | SSE-6  Go back to sleep within 15 minutes of waking in the night. | SSE-7  Feel refreshed upon waking in the morning. | SSE-8  Wake after a poor night's sleep without feeling upset about it. | SSE-9  Not allow a poor night's sleep to interfere with daily activities. |
| --- | --- | --- | --- | --- | --- | --- | --- | --- | --- |
| Mental demand | | | | | | | | | |
| r | .164 | .216 | -.100 | .017 | -.137 | -.049 | .115 | -.084 | -.309 |
| P-value  (two-tailed test) | .363 | .227 | .581 | .926 | .446 | .789 | .524 | .644 | .080 |
| Physical demand | | | | | | | | | |
| r | -.152 | -.246 | -.055 | -.129 | -.266 | -.327 | .015 | -.152 | -.010 |
| P-value  (two-tailed test) | .397 | .168 | .761 | .475 | .135 | .063 | .933 | .397 | .957 |
| Temporal demand | | | | | | | | | |
| r | .089 | -.193 | -.329 | -.057 | -.122 | .036 | -.025 | -.381 | -.070 |
| P-value  (two-tailed test) | .624 | .282 | .061 | .755 | .499 | .842 | .890 | .028 | .699 |
| Performance | | | | | | | | | |
| r | .120 | -.036 | -.442 | -.035 | -.103 | -.166 | -.009 | -.136 | -.233 |
| P-value  (two-tailed test) | .507 | .844 | .010 | .845 | .567 | .357 | .960 | .450 | .192 |
| Effort | | | | | | | | | |
| r | .014 | -.104 | -.415 | -.120 | -.028 | -.004 | -.101 | -.292 | -.274 |
| P-value  (two-tailed test) | .939 | .563 | .016 | .506 | .876 | .984 | .577 | .099 | .123 |
| Frustration | | | | | | | | | |
| r | .005 | -.020 | -.163 | -.044 | -.129 | -.191 | -.017 | -.078 | -.260 |
| P-value  (two-tailed test) | .976 | .912 | .366 | .807 | .476 | .287 | .925 | .667 | .144 |
| NASA-TLX total | | | | | | | | | |
| r | .055 | -.098 | -.345 | -.085 | -.178 | -.147 | -.008 | -.273 | -.256 |
| P-value  (two-tailed test) | .762 | .589 | .049 | .636 | .321 | .414 | .967 | .125 | .150 |

1. **Correlations among sleep self-efficacy and gaming experience for four interfaces.**

Interface1 (Countdown)

| Variable | SSE-1  Lie in bed, feeling physically relaxed. | SSE-2  Lie in bed, feeling mentally relaxed. | SSE-3  Lie in bed with your thoughts "turned off". | SSE-4  Fall asleep at night in under 30 minutes. | SSE-5  Wake up at night fewer than 3 times. | SSE-6  Go back to sleep within 15 minutes of waking in the night. | SSE-7  Feel refreshed upon waking in the morning. | SSE-8  Wake after a poor night's sleep without feeling upset about it. | SSE-9  Not allow a poor night's sleep to interfere with daily activities. |
| --- | --- | --- | --- | --- | --- | --- | --- | --- | --- |
| Competence | | | | | | | | | |
| r | -.030 | -.066 | .055 | -.233 | .039 | -.010 | .372 | .233 | .276 |
| P-value  (two-tailed test) | .867 | .716 | .760 | .191 | .829 | .956 | .033 | .192 | .120 |
| Immersion | | | | | | | | | |
| r | .267 | .269 | .243 | .149 | .169 | .133 | .458 | .427 | .241 |
| P-value  (two-tailed test) | .133 | .131 | .174 | .407 | .347 | .460 | .007 | .013 | .177 |
| Flow | | | | | | | | | |
| r | -.102 | -.053 | .282 | .042 | -.240 | .018 | .255 | .160 | .146 |
| P-value  (two-tailed test) | .571 | .770 | .112 | .815 | .179 | .921 | .152 | .375 | .419 |
| Tension | | | | | | | | | |
| r | -.230 | -.016 | .168 | .162 | .133 | -.109 | -.383 | -.196 | -.210 |
| P-value  (two-tailed test) | .463 | .561 | .344 | .115 | .720 | .419 | .457 | .891 | .301 |
| Challenge | | | | | | | | | |
| r | -.083 | -.106 | -.059 | .080 | .040 | -.194 | -.099 | -.082 | -.097 |
| P-value  (two-tailed test) | .646 | .556 | .743 | .658 | .825 | .280 | .582 | .649 | .589 |
| Negative affect | | | | | | | | | |
| r | .120 | .180 | -.138 | .300 | .133 | -.135 | -.074 | .118 | -.122 |
| P-value  (two-tailed test) | .506 | .317 | .442 | .090 | .460 | .454 | .682 | .514 | .497 |
| Positive affect | | | | | | | | | |
| r | -.110 | -.132 | -.209 | .082 | -.074 | -.105 | -.189 | -.208 | -.164 |
| P-value  (two-tailed test) | .541 | .463 | .244 | .649 | .682 | .561 | .293 | .245 | .361 |

Interface2 (Zoom in/out)

| Variable | SSE-1  Lie in bed, feeling physically relaxed. | SSE-2  Lie in bed, feeling mentally relaxed. | SSE-3  Lie in bed with your thoughts "turned off". | SSE-4  Fall asleep at night in under 30 minutes. | SSE-5  Wake up at night fewer than 3 times. | SSE-6  Go back to sleep within 15 minutes of waking in the night. | SSE-7  Feel refreshed upon waking in the morning. | SSE-8  Wake after a poor night's sleep without feeling upset about it. | SSE-9  Not allow a poor night's sleep to interfere with daily activities. |
| --- | --- | --- | --- | --- | --- | --- | --- | --- | --- |
| Competence | | | | | | | | | |
| r | .034 | .128 | .097 | .133 | .158 | .348 | .130 | .260 | .111 |
| P-value  (two-tailed test) | .852 | .477 | .591 | .460 | .380 | .047 | .470 | .144 | .538 |
| Immersion | | | | | | | | | |
| r | .218 | .328 | .472 | .363 | .187 | -.075 | .434 | .308 | -.019 |
| P-value  (two-tailed test) | .224 | .062 | .006 | .038 | .297 | .678 | .012 | .081 | .916 |
| Flow | | | | | | | | | |
| r | -.140 | .003 | .189 | -.002 | -.308 | -.026 | .157 | .055 | .091 |
| P-value  (two-tailed test) | .438 | .985 | .293 | .992 | .081 | .885 | .384 | .761 | .615 |
| Tension | | | | | | | | | |
| r | -.265 | -.105 | .271 | .044 | .022 | -.335 | -.252 | -.251 | -.119 |
| P-value  (two-tailed test) | .136 | .559 | .127 | .807 | .905 | .057 | .156 | .159 | .511 |
| Challenge | | | | | | | | | |
| r | -.139 | -.199 | .014 | -.063 | -.091 | -.233 | -.297 | -.421 | -.123 |
| P-value  (two-tailed test) | .441 | .266 | .938 | .728 | .613 | .193 | .093 | .015 | .494 |
| Negative affect | | | | | | | | | |
| r | .020 | -.051 | .109 | .078 | .287 | .071 | -.356 | -.231 | -.085 |
| P-value  (two-tailed test) | .913 | .780 | .548 | .665 | .105 | .693 | .042 | .196 | .638 |
| Positive affect | | | | | | | | | |
| r | -.100 | .101 | .220 | .035 | -.043 | .027 | .348 | .176 | -.060 |
| P-value  (two-tailed test) | .582 | .576 | .219 | .846 | .811 | .883 | .047 | .328 | .742 |

Interface3 (Up/down)

| Variable | SSE-1  Lie in bed, feeling physically relaxed. | SSE-2  Lie in bed, feeling mentally relaxed. | SSE-3  Lie in bed with your thoughts "turned off". | SSE-4  Fall asleep at night in under 30 minutes. | SSE-5  Wake up at night fewer than 3 times. | SSE-6  Go back to sleep within 15 minutes of waking in the night. | SSE-7  Feel refreshed upon waking in the morning. | SSE-8  Wake after a poor night's sleep without feeling upset about it. | SSE-9  Not allow a poor night's sleep to interfere with daily activities. |
| --- | --- | --- | --- | --- | --- | --- | --- | --- | --- |
| Competence | | | | | | | | | |
| r | .096 | .159 | .071 | .005 | .322 | .137 | .312 | .384 | .400 |
| P-value  (two-tailed test) | .594 | .375 | .694 | .980 | .068 | .447 | .077 | .027 | .021 |
| Immersion | | | | | | | | | |
| r | -.116 | .109 | .303 | .086 | .194 | -.063 | .221 | .164 | .197 |
| P-value  (two-tailed test) | .519 | .546 | .087 | .634 | .280 | .727 | .216 | .361 | .271 |
| Flow | | | | | | | | | |
| r | -.010 | -.045 | -.124 | -.129 | -.265 | .144 | .153 | .027 | .139 |
| P-value  (two-tailed test) | .956 | .804 | .492 | .473 | .136 | .426 | .395 | .883 | .441 |
| Tension | | | | | | | | | |
| r | -.086 | .093 | .497 | .152 | .214 | -.180 | -.064 | .018 | -.247 |
| P-value  (two-tailed test) | .633 | .605 | .003 | .398 | .232 | .315 | .723 | .919 | .165 |
| Challenge | | | | | | | | | |
| r | .036 | .081 | .190 | .035 | -.006 | -.046 | -.130 | -.217 | -.218 |
| P-value  (two-tailed test) | .842 | .655 | .290 | .849 | .972 | .797 | .472 | .225 | .224 |
| Negative affect | | | | | | | | | |
| r | -.044 | .119 | .332 | .143 | .325 | .066 | -.194 | .044 | -.363 |
| P-value  (two-tailed test) | .809 | .508 | .059 | .429 | .065 | .715 | .279 | .807 | .038 |
| Positive affect | | | | | | | | | |
| r | .001 | .135 | .033 | .017 | .052 | .073 | .252 | .114 | .166 |
| P-value  (two-tailed test) | .997 | .453 | .855 | .925 | .772 | .686 | .156 | .527 | .356 |

Interface4 (Color gradients)

| Variable | SSE-1  Lie in bed, feeling physically relaxed. | SSE-2  Lie in bed, feeling mentally relaxed. | SSE-3  Lie in bed with your thoughts "turned off". | SSE-4  Fall asleep at night in under 30 minutes. | SSE-5  Wake up at night fewer than 3 times. | SSE-6  Go back to sleep within 15 minutes of waking in the night. | SSE-7  Feel refreshed upon waking in the morning. | SSE-8  Wake after a poor night's sleep without feeling upset about it. | SSE-9  Not allow a poor night's sleep to interfere with daily activities. |
| --- | --- | --- | --- | --- | --- | --- | --- | --- | --- |
| Competence | | | | | | | | | |
| r | .161 | .221 | .198 | .015 | .130 | .190 | .180 | .283 | .196 |
| P-value  (two-tailed test) | .372 | .217 | .270 | .936 | .471 | .290 | .317 | .111 | .274 |
| Immersion | | | | | | | | | |
| r | .297 | .255 | .281 | .118 | -.064 | -.039 | .567 | .368 | .191 |
| P-value  (two-tailed test) | .093 | .153 | .113 | .514 | .723 | .831 | .001 | .035 | .287 |
| Flow | | | | | | | | | |
| r | -.021 | .014 | .151 | -.110 | -.222 | .154 | .235 | .290 | .154 |
| P-value  (two-tailed test) | .909 | .940 | .402 | .543 | .214 | .392 | .187 | .102 | .392 |
| Tension | | | | | | | | | |
| r | -.153 | -.049 | -.012 | .224 | .185 | .019 | -.246 | -.148 | -.242 |
| P-value  (two-tailed test) | .397 | .788 | .948 | .211 | .303 | .917 | .168 | .412 | .174 |
| Challenge | | | | | | | | | |
| r | -.087 | -.096 | -.044 | .116 | .112 | -.115 | -.067 | -.166 | -.084 |
| P-value  (two-tailed test) | .629 | .596 | .810 | .520 | .533 | .524 | .711 | .356 | .642 |
| Negative affect | | | | | | | | | |
| r | .055 | .128 | .062 | .256 | .393 | .111 | -.008 | .031 | -.126 |
| P-value  (two-tailed test) | .761 | .479 | .732 | .151 | .024 | .540 | .966 | .862 | .486 |
| Positive affect | | | | | | | | | |
| r | .230 | .249 | .213 | -.055 | -.155 | .000 | .290 | .187 | .103 |
| P-value  (two-tailed test) | .197 | .163 | .234 | .762 | .390 | 1.000 | .102 | .297 | .569 |

1. **Correlations among physiological/psychological signals and cognitive load for four interfaces.**

Interface1 (Countdown)

| Variable | Breathing rate | Mean heart rate | Mean SDNN | Mean RMSSD | Relaxation | Relaxation stability | Attention | Attention stability |
| --- | --- | --- | --- | --- | --- | --- | --- | --- |
| Mental demand | | | | | | | | |
| r | .246 | -.158 | .210 | .091 | .155 | .054 | -.207 | -.073 |
| P-value  (two-tailed test) | .174 | .381 | .242 | .616 | .389 | .766 | .248 | .685 |
| Physical demand | | | | | | | | |
| r | .058 | -.085 | .141 | -.027 | -.051 | .151 | .107 | -.118 |
| P-value  (two-tailed test) | .751 | .637 | .434 | .879 | .776 | .401 | .553 | .514 |
| Temporal demand | | | | | | | | |
| r | .188 | -.234 | .198 | .066 | .334 | -.045 | .128 | -.135 |
| P-value  (two-tailed test) | .302 | .190 | .270 | .713 | .057 | .804 | .476 | .455 |
| Performance | | | | | | | | |
| r | .006 | -.385 | .385 | .342 | -.096 | .163 | -.113 | -.080 |
| P-value  (two-tailed test) | .972 | .027 | .027 | .051 | .597 | .366 | .531 | .658 |
| Effort | | | | | | | | |
| r | -.025 | -.305 | .330 | .271 | -.060 | .073 | -.002 | .035 |
| P-value  (two-tailed test) | .893 | .084 | .061 | .127 | .739 | .685 | .991 | .845 |
| Frustration | | | | | | | | |
| r | -.016 | -.245 | .306 | .307 | .185 | -.097 | .097 | .024 |
| P-value  (two-tailed test) | .929 | .169 | .084 | .082 | .304 | .592 | .593 | .897 |
| NASA-TLX total | | | | | | | | |
| r | .113 | -.332 | .368 | .237 | .133 | .063 | .024 | -.090 |
| P-value  (two-tailed test) | .538 | .059 | .035 | .183 | .462 | .727 | .895 | .619 |

Interface2 (Zoom in/out)

| Variable | Breathing rate | Mean heart rate | Mean SDNN | Mean RMSSD | Relaxation | Relaxation stability | Attention | Attention stability |
| --- | --- | --- | --- | --- | --- | --- | --- | --- |
| Mental demand | | | | | | | | |
| r | .030 | -.036 | .103 | -.081 | -.073 | -.062 | -.377 | .427 |
| P-value  (two-tailed test) | .869 | .842 | .569 | .652 | .684 | .730 | .031 | .013 |
| Physical demand | | | | | | | | |
| r | -.090 | .036 | -.018 | -.077 | -.050 | -.053 | -.047 | .290 |
| P-value  (two-tailed test) | .623 | .843 | .921 | .669 | .784 | .771 | .794 | .102 |
| Temporal demand | | | | | | | | |
| r | .269 | .021 | .005 | -.031 | .156 | -.217 | -.350 | .191 |
| P-value  (two-tailed test) | .136 | .909 | .978 | .863 | .385 | .224 | .046 | .286 |
| Performance | | | | | | | | |
| r | .178 | .149 | -.129 | -.073 | -.044 | -.248 | -.120 | .353 |
| P-value  (two-tailed test) | .329 | .407 | .475 | .688 | .808 | .163 | .504 | .044 |
| Effort | | | | | | | | |
| r | -.169 | -.047 | .119 | .100 | -.093 | -.186 | -.058 | .539 |
| P-value  (two-tailed test) | .354 | .793 | .508 | .578 | .606 | .299 | .748 | .001 |
| Frustration | | | | | | | | |
| r | .132 | -.052 | .250 | .274 | -.109 | -.088 | -.059 | .458 |
| P-value  (two-tailed test) | .472 | .773 | .161 | .123 | .546 | .626 | .746 | .007 |
| NASA-TLX total | | | | | | | | |
| r | .077 | .013 | .076 | .027 | -.045 | -.186 | -.222 | .496 |
| P-value  (two-tailed test) | .676 | .941 | .676 | .883 | .803 | .300 | .215 | .003 |

Interface3 (Up/down)

| Variable | Breathing rate | Mean heart rate | Mean SDNN | Mean RMSSD | Relaxation | Relaxation stability | Attention | Attention stability |
| --- | --- | --- | --- | --- | --- | --- | --- | --- |
| Mental demand | | | | | | | | |
| r | -.190 | -.151 | .184 | .217 | .070 | .257 | -.153 | .112 |
| P-value  (two-tailed test) | .298 | .401 | .305 | .225 | .699 | .149 | .396 | .536 |
| Physical demand | | | | | | | | |
| r | -.126 | -.139 | .255 | .089 | .329 | .122 | -.038 | -.132 |
| P-value  (two-tailed test) | .492 | .442 | .153 | .621 | .061 | .498 | .834 | .465 |
| Temporal demand | | | | | | | | |
| r | .076 | -.221 | .205 | .314 | .058 | .043 | -.019 | .182 |
| P-value  (two-tailed test) | .681 | .217 | .253 | .076 | .749 | .813 | .917 | .311 |
| Performance | | | | | | | | |
| r | -.130 | -.194 | .111 | .177 | .110 | -.052 | -.214 | -.034 |
| P-value  (two-tailed test) | .479 | .280 | .539 | .323 | .542 | .775 | .231 | .853 |
| Effort | | | | | | | | |
| r | -.118 | -.236 | .337 | .146 | .392 | .179 | -.074 | -.066 |
| P-value  (two-tailed test) | .520 | .185 | .055 | .417 | .024 | .318 | .683 | .714 |
| Frustration | | | | | | | | |
| r | -.054 | -.073 | .063 | .119 | -.012 | -.122 | -.244 | -.190 |
| P-value  (two-tailed test) | .771 | .685 | .727 | .511 | .945 | .500 | .170 | .290 |
| NASA-TLX total | | | | | | | | |
| r | -.113 | -.222 | .260 | .231 | .218 | .107 | -.147 | -.020 |
| P-value  (two-tailed test) | .538 | .214 | .143 | .197 | .223 | .555 | .415 | .913 |

Interface4 (Color gradients)

| Variable | Breathing rate | Mean heart rate | Mean SDNN | Mean RMSSD | Relaxation | Relaxation stability | Attention | Attention stability |
| --- | --- | --- | --- | --- | --- | --- | --- | --- |
| Mental demand | | | | | | | | |
| r | .232 | .072 | .181 | .070 | -.009 | -.116 | -.493 | .086 |
| P-value  (two-tailed test) | .201 | .689 | .313 | .699 | .961 | .520 | .004 | .636 |
| Physical demand | | | | | | | | |
| r | .256 | -.195 | .389 | .239 | .342 | .358 | -.248 | .202 |
| P-value  (two-tailed test) | .157 | .277 | .025 | .181 | .051 | .041 | .164 | .261 |
| Temporal demand | | | | | | | | |
| r | .413 | .380 | -.077 | -.195 | .107 | .094 | -.142 | .405 |
| P-value  (two-tailed test) | .019 | .029 | .668 | .278 | .553 | .602 | .431 | .019 |
| Performance | | | | | | | | |
| r | .150 | .217 | -.006 | .023 | .076 | .001 | -.248 | .351 |
| P-value  (two-tailed test) | .412 | .224 | .973 | .900 | .672 | .994 | .163 | .045 |
| Effort | | | | | | | | |
| r | .201 | -.067 | .258 | .285 | .005 | .144 | -.266 | .336 |
| P-value  (two-tailed test) | .270 | .709 | .147 | .108 | .979 | .424 | .135 | .056 |
| Frustration | | | | | | | | |
| r | .130 | .188 | .090 | .173 | .175 | -.028 | -.224 | .191 |
| P-value  (two-tailed test) | .478 | .293 | .620 | .336 | .331 | .877 | .211 | .287 |
| NASA-TLX total | | | | | | | | |
| r | .336 | .149 | .183 | .122 | .159 | .108 | -.362 | .367 |
| P-value  (two-tailed test) | .060 | .408 | .309 | .499 | .378 | .549 | .038 | .036 |

1. **Correlations among physiological/psychological signals and cognitive load for four interfaces.**

Interface1 (Countdown)

| Variable | Breathing rate | Mean heart rate | Mean SDNN | Mean RMSSD | Relaxation | Relaxation stability | Attention | Attention stability |
| --- | --- | --- | --- | --- | --- | --- | --- | --- |
| Competence | | | | | | | | |
| r | -.252 | .237 | -.301 | -.278 | -.152 | .189 | -.076 | -.043 |
| P-value  (two-tailed test) | .164 | .183 | .089 | .118 | .399 | .292 | .674 | .814 |
| Immersion | | | | | | | | |
| r | .160 | .125 | -.283 | -.082 | -.011 | -.154 | -.067 | .234 |
| P-value  (two-tailed test) | .381 | .489 | .111 | .650 | .950 | .393 | .711 | .190 |
| Flow | | | | | | | | |
| r | .005 | .115 | -.187 | -.061 | -.052 | -.109 | -.077 | .187 |
| P-value  (two-tailed test) | .979 | .524 | .296 | .736 | .774 | .547 | .672 | .296 |
| Tension | | | | | | | | |
| r | .203 | -.217 | .214 | .181 | .239 | -.314 | .039 | -.003 |
| P-value  (two-tailed test) | .265 | .226 | .231 | .314 | .180 | .075 | .828 | .986 |
| Challenge | | | | | | | | |
| r | .378 | -.337 | .307 | .217 | .268 | -.302 | .226 | .095 |
| P-value  (two-tailed test) | **.033** | .055 | .083 | .224 | .132 | .088 | .205 | .599 |
| Negative affect | | | | | | | | |
| r | .259 | -.289 | .315 | .281 | .219 | -.277 | .164 | -.012 |
| P-value  (two-tailed test) | .152 | .103 | .074 | .113 | .220 | .119 | .361 | .945 |
| Positive affect | | | | | | | | |
| r | -.040 | .239 | -.314 | -.290 | -.091 | .018 | .064 | .128 |
| P-value  (two-tailed test) | .829 | .180 | .075 | .101 | .613 | .921 | .724 | .477 |

Interface2 (Zoom in/out)

| Variable | Breathing rate | Mean heart rate | Mean SDNN | Mean RMSSD | Relaxation | Relaxation stability | Attention | Attention stability |
| --- | --- | --- | --- | --- | --- | --- | --- | --- |
| Competence | | | | | | | | |
| r | .062 | .193 | -.220 | -.241 | .079 | .066 | .064 | -.261 |
| P-value  (two-tailed test) | .736 | .283 | .218 | .176 | .661 | .717 | .724 | .142 |
| Immersion | | | | | | | | |
| r | -.045 | .140 | -.090 | -.158 | -.101 | .297 | .003 | -.226 |
| P-value  (two-tailed test) | .805 | .437 | .617 | .381 | .575 | .093 | .986 | .206 |
| Flow | | | | | | | | |
| r | -.158 | .123 | -.050 | -.241 | .059 | .327 | -.044 | -.031 |
| P-value  (two-tailed test) | .387 | .495 | .784 | .177 | .745 | .063 | .809 | .862 |
| Tension | | | | | | | | |
| r | .033 | .067 | .072 | .138 | .105 | -.081 | .047 | .140 |
| P-value  (two-tailed test) | .856 | .710 | .689 | .442 | .562 | .655 | .793 | .438 |
| Challenge | | | | | | | | |
| r | .090 | -.007 | .070 | .176 | -.049 | -.092 | -.023 | .429 |
| P-value  (two-tailed test) | .623 | .971 | .699 | .326 | .786 | .611 | .900 | .013 |
| Negative affect | | | | | | | | |
| r | -.051 | -.327 | .256 | .442 | .052 | -.183 | -.143 | -.054 |
| P-value  (two-tailed test) | .780 | .064 | .150 | .010 | .774 | .307 | .428 | .764 |
| Positive affect | | | | | | | | |
| r | -.006 | .261 | -.259 | -.395 | -.036 | .315 | .047 | -.181 |
| P-value  (two-tailed test) | .974 | .142 | .145 | .023 | .844 | .074 | .795 | .314 |

Interface3 (Up/down)

| Variable | Breathing rate | Mean heart rate | Mean SDNN | Mean RMSSD | Relaxation | Relaxation stability | Attention | Attention stability |
| --- | --- | --- | --- | --- | --- | --- | --- | --- |
| Competence | | | | | | | | |
| r | -.221 | .173 | -.245 | -.391 | -.161 | -.111 | .119 | .188 |
| P-value  (two-tailed test) | .225 | .335 | .170 | .024 | .371 | .539 | .510 | .294 |
| Immersion | | | | | | | | |
| r | -.054 | .123 | -.153 | -.339 | -.307 | -.348 | .251 | .081 |
| P-value  (two-tailed test) | .771 | .496 | .394 | .053 | .082 | .047 | .159 | .654 |
| Flow | | | | | | | | |
| r | -.128 | .077 | -.052 | -.216 | -.030 | .102 | -.023 | .081 |
| P-value  (two-tailed test) | .486 | .669 | .775 | .228 | .867 | .572 | .897 | .654 |
| Tension | | | | | | | | |
| r | .249 | -.028 | .118 | .234 | .033 | -.281 | -.292 | -.182 |
| P-value  (two-tailed test) | .169 | .875 | .511 | .190 | .856 | .113 | .099 | .310 |
| Challenge | | | | | | | | |
| r | .387 | -.075 | .169 | .178 | .042 | -.358 | .002 | -.088 |
| P-value  (two-tailed test) | .029 | .676 | .346 | .321 | .816 | .041 | .992 | .627 |
| Negative affect | | | | | | | | |
| r | .213 | -.200 | .103 | .384 | .092 | -.198 | -.244 | -.253 |
| P-value  (two-tailed test) | .241 | .265 | .568 | .027 | .611 | .269 | .172 | .155 |
| Positive affect | | | | | | | | |
| r | -.112 | .152 | -.147 | -.385 | -.178 | -.166 | .199 | .234 |
| P-value  (two-tailed test) | .541 | .397 | .414 | .027 | .321 | .356 | .267 | .189 |

Interface4 (Color gradients)

| Variable | Breathing rate | Mean heart rate | Mean SDNN | Mean RMSSD | Relaxation | Relaxation stability | Attention | Attention stability |
| --- | --- | --- | --- | --- | --- | --- | --- | --- |
| Competence | | | | | | | | |
| r | -.138 | -.219 | .001 | -.060 | .078 | .087 | .069 | -.327 |
| P-value  (two-tailed test) | .451 | .220 | .995 | .740 | .668 | .632 | .704 | .063 |
| Immersion | | | | | | | | |
| r | -.140 | .145 | -.232 | -.248 | .080 | .103 | -.012 | -.227 |
| P-value  (two-tailed test) | .446 | .421 | .194 | .165 | .657 | .568 | .948 | .204 |
| Flow | | | | | | | | |
| r | -.053 | -.303 | .297 | .214 | .053 | .146 | -.142 | -.304 |
| P-value  (two-tailed test) | .774 | .087 | .093 | .231 | .771 | .418 | .431 | .085 |
| Tension | | | | | | | | |
| r | .435 | .313 | -.265 | -.093 | .140 | -.217 | .158 | .235 |
| P-value  (two-tailed test) | .013 | .076 | .136 | .607 | .436 | .225 | .380 | .187 |
| Challenge | | | | | | | | |
| r | .430 | .172 | -.146 | -.046 | .202 | .134 | .286 | .296 |
| P-value  (two-tailed test) | .014 | .337 | .419 | .801 | .259 | .456 | .106 | .094 |
| Negative affect | | | | | | | | |
| r | .281 | .097 | -.145 | .006 | -.070 | -.135 | -.065 | .098 |
| P-value  (two-tailed test) | .120 | .590 | .420 | .973 | .698 | .455 | .718 | .589 |
| Positive affect | | | | | | | | |
| r | -.137 | -.013 | -.043 | -.145 | .153 | .094 | .108 | -.341 |
| P-value  (two-tailed test) | .455 | .944 | .812 | .421 | .395 | .601 | .549 | .052 |
